# Stressed, Stained, and Stored: The Impact of Maternal Distress and Smoking on Amniotic Biomarkers—A Scoping Review

**DOI:** 10.3390/ijms27188434

**Published:** 2026-09-21

**Authors:** Krystian Skowron, Filip Godziszewski, Anna Putyrska, Alicja Łyczkowska, Nikodem Skrzypiec, Magdalena Orczyk-Pawiłowicz

**Affiliations:** 1 Scientific Association of Medical Chemistry and Immunochemistry, Wroclaw Medical University, 50-369 Wroclaw, Poland; 2 Student Scientific Association for Children’s Surgery, Wroclaw Medical University, 50-369 Wroclaw, Poland; anna.putyrska@student.umw.edu.pl (A.P.); alicja.lyczkowska@student.umw.edu.pl (A.Ł.); nikodem.skrzypiec@student.umw.edu.pl (N.S.); 3 Clinical and Dissecting Anatomy Students’ Scientific Club, Wroclaw Medical University, 50-369 Wroclaw, Poland; filip.godziszewski@student.umw.edu.pl; 4 Student Scientific Association of Neurology, Wroclaw Medical University, 50-369 Wroclaw, Poland; 5 Division of Chemistry and Immunochemistry, Department of Biochemistry and Immunochemistry, Wroclaw Medical University, 50-369 Wroclaw, Poland

**Keywords:** amniotic fluid, psychological factors, smoking, selective serotonin reuptake inhibitors, maternal lifestyle

## Abstract

This scoping review represents a comprehensive effort to conceptualize the mother, fetus, and amniotic fluid as a dynamic triad. The primary objective is to map the available literature and synthesize findings regarding how maternal mental health, psychiatric treatments, and environmental lifestyle exposures (specifically smoking) affect the biochemical composition of amniotic fluid in the context of metabolic programming. A systematic search was conducted using PubMed, Web of Science, and Embase in accordance with PRISMA Extension for Scoping Reviews guidelines. The quality (using the Quality Assessment Tool for Observational Cohort and Cross-Sectional Studies designed by the NIH) of all 30 original articles was assessed, and data regarding pregnancy and amniotic fluid findings were extracted for narrative synthesis. Chronic stress and maternal anxiety positively correlate with cortisol and corticotropin-releasing hormone levels in amniotic fluid. Psychiatric treatment with selective serotonin reuptake inhibitors was linked to elevated neuronal injury markers, such as S100B and activin A. Furthermore, smoking during pregnancy interferes with amniotic metabolic pathways, including those related to oxidative stress. The available evidence, despite limitations in examining amniotic fluid and the challenges of scientifically measuring maternal stress, supports the role of a dynamic triad between the mother, amniotic fluid, and the fetus and its interface in shaping fetal development and long-term health outcomes. Changes at the biochemical, hormonal, and genetic levels in amniotic fluid composition support the concept of metabolic programming. Understanding these pathways is crucial for recognizing early influences on fetal development and optimizing long-term health outcomes.

## 1. Introduction

Amniotic fluid (AF) provides a physical and biochemical environment that allows the fetus to grow and develop in precisely regulated conditions. The volume of AF is the result of an equilibrium between the mother, fetus, and placenta and, in a healthy pregnancy, falls within a narrow range [1]. The biochemical composition of the AF reflects multiple functions. Apart from providing mechanical protection, AF serves as a source of crucial growth factors, vitamins, ions and structural compounds (proteins), particularly during the development of the musculoskeletal, gastrointestinal, and pulmonary systems [2,3]. Fetal development is a dynamic process; thus, the composition of AF must undergo changes. At the beginning, AF consists mainly of water and electrolytes, while after 12–14 weeks of gestation, additional compounds such as hormones, lipids, proteins, and carbohydrates are also present [4]. It is associated with changes in the formation of AF. During the first trimester, it is a transudate of plasma, which may originate from the mother (across the uterine decidua or directly from the placenta) or fetus. Placental transport, occurring through paracellular and transcellular routes (including transtrophoblastic channels), regulates the movement of solutes and water to define the biochemical composition of the amniotic fluid. This barrier allows for the passage of lipid-soluble molecules and small hydrophilic substances, with water transfer further facilitated by specialized membrane proteins known as aquaporins [5]. Initially, fetal skin is permeable, allowing participation in the AF equilibrium; however, keratinization, occurring between the 20th and the 25th weeks of pregnancy, ceases this process. With pregnancy progression, AF composition shifts from plasma-like to a fluid with lower osmolality and a higher content of fetal uric compounds. Subsequently, AF is swallowed and excreted by the fetus, which also allows AF to play a role in organogenesis and potentially influence this sophisticated process [6,7]. The trophic effect that AF has on fetal intestinal epithelial cells has been considered comparable to that of breast milk, which is another crucial fluid in offspring development and maturation [8]. Studies also show how exposure to certain chemicals via AF may shape child development even after birth [9]. Additionally, AF facilitates protection from infections due to the presence of antibodies and antibacterial compounds [10].

Metabolic programming has been defined as a process where early-life stimuli permanently alter an organism by inducing, deleting, or disrupting the development of somatic structures, or by influencing the foundational settings of physiological systems. Amniotic fluid may serve as a tool for time-specific monitoring of fetal development and metabolism, including endo- and exogenous substances circulating between mother and fetus. Recent metabolomics studies have the potential to monitor diet-induced metabolic signals or exposure to certain substances [11].

The examination of AF is a challenging subject, and there are still gaps in understanding of this important gestational factor. This is largely due to difficulties in material retrieval. Amniocentesis is performed in humans around 16–20 weeks of gestation in order to perform clinical and genetic testing. The procedure is usually not repeated, which limits the ability to track changes in amniotic composition throughout pregnancy, as well as the impact of the maternal environment and factors on shaping AF composition [12]. Technological developments, mainly ultrasound, may facilitate easier AF collection. While some studies point out possible complications like bleeding, amniotic fluid leakage or even miscarriage [13], other studies conducted at a similar time but in a different country emphasize minimal risk of the procedure in comparison to possible medical benefits. Authors point out that miscarriage and preterm delivery commonly attributed to amniocentesis do not occur statistically more frequently in women who undergo the procedure [14].

**Figure 1:**
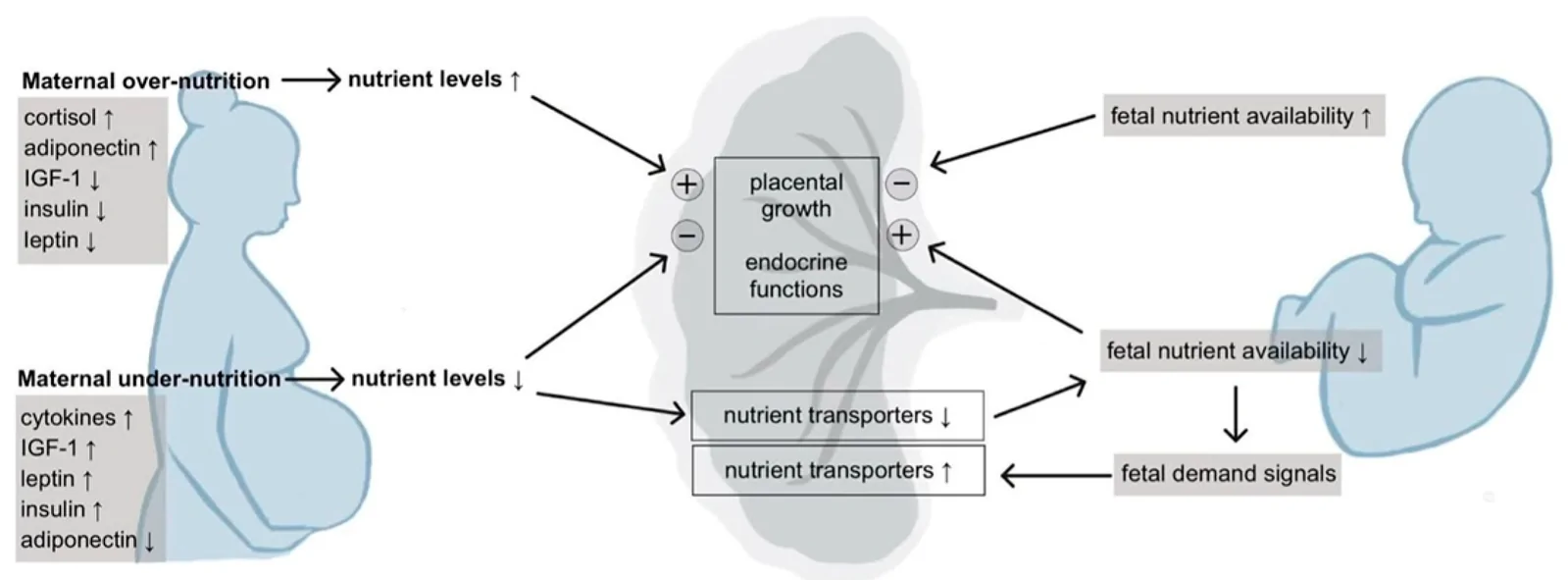
Maternal–placental–fetal triad in fetal demand and nutrient-sensing models (adapted from [19]).

Despite these challenges, researchers in recent years have contributed a great deal of added information about AF. By pairing samples obtained from rhesus macaques with human-derived samples, insight into the AF proteome has been gained [6]. Additionally, there are studies identifying maternal factors that may change AF composition, thus potentially influencing in utero fetal development. Regarding maternal factors with suspected influence on the composition of amniotic fluid, lifestyle choices and diet have been investigated. Alterations may be associated with nutrition [15] or metabolic conditions such as gestational diabetes mellitus [16,17]. The placenta functions as a dynamic sensor that adjusts the transport of amino acids, glucose, and fatty acids by integrating maternal nutritional cues and fetal demand signals to regulate resource allocation (Figure 1). By modulating these transport activities, the placenta balances fetal growth with maternal supply capacity, thereby playing a critical role in fetal programming and the long-term health of the offspring [18,19].

Stress during pregnancy is a key factor in the mother–child dyad dynamic reflected via fluids like breast milk or amniotic fluid [20]. It affects the hypothalamic–pituitary–adrenal axis, which is most associated with stress, because the organism is a complex, interconnected system; other metabolic pathways may also be involved [21,22,23]. During severe stress or conditions like depression, low-grade inflammation may be present and alter the mother–fetus–amniotic fluid equilibrium by influencing placental function [16,24]. Similar biological changes have been observed in women experiencing anxiety or stress [25]. With severe conditions, like depression, the question of in utero drug exposure arises. Loughhead et al. reported that AF is another route of exposure for the fetus, while knowledge is still lacking about the outcome of AF-mediated fetal exposure to antidepressant drugs [26]. Indirect alterations in the amniotic fluid profile may be driven by maladaptive maternal behaviors, such as substance misuse or nutritional deficiencies, which are frequently associated with compromised antenatal mental health [27]. Additionally, prenatal alcohol exposure has been identified as a serious threat that may result in fetal alcohol spectrum disorders (FASDs) or even the death of a child. The fetus is especially vulnerable to harmful xenobiotics due to reduced metabolic capacity and prolonged exposure via constantly circulating amniotic fluid [28]. A rodent model also showed the effect of intrauterine exposure to nicotine; studies provide evidence for dose-dependent alteration in immune and inflammatory responses, which can influence fetal development and programming [29]. Similar to FASD, intrauterine infection (which may be a result of smoking during pregnancy) may be linked with adverse health outcomes later in life, the best known of which are TORCH infections [30]. In light of the above, this review aims to consolidate current findings regarding the impact of maternal stress, mental health disorders, and associated lifestyle habits on the biochemical profile of amniotic fluid. Furthermore, the potential impact of these alterations on fetal well-being and long-term outcomes is discussed. To the best of our knowledge, this is the first review exploring this type of maternal–fetal connection.

## 2. Materials and Methods

### 2.1. Study Design and Research Question

Preliminary investigations were undertaken to refine the research scope and delineate the specific parameters and variables requisite for the literature search. The aim was to synthesize current knowledge regarding the relationship between maternal mental state (directly or via behavioral determinants) and amniotic fluid composition. The study protocol was not registered.

### 2.2. Search Strategy, Eligibility Criteria, Study Selection

A systematic search of PubMed, Web of Science, and Embase was conducted using a Boolean search strategy. English-language original articles that investigated changes in amniotic fluid properties in response to stress-related factors were sought. Publications were included if they either assessed mental state (by well-established questionnaires or formal psychiatric diagnosis) or examined factors that are claimed to be linked with deteriorating mental status (and the claim is confirmed by available literature). During the PubMed search, the “human-only” filter was imposed. No publication-date restrictions were applied, as this is the first review on the topic.

**Figure 2:**
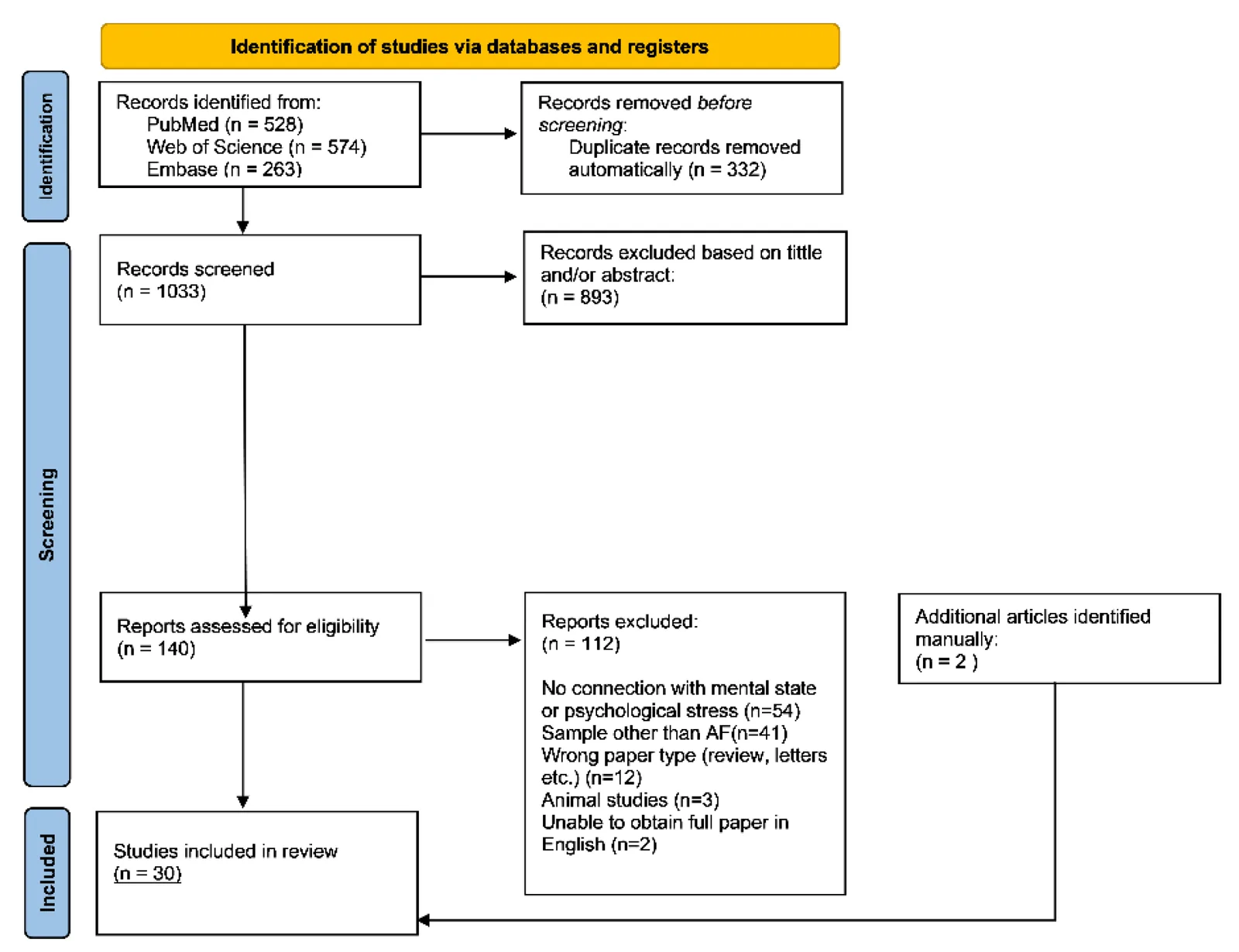
PRISMA flowchart [32].

As illustrated in the flow diagram, the final synthesis comprises 30 articles published through 16 March 2026 (initial search was performed on 11 November 2025, while an updated search for newly published studies took place on 16 March 2026). Data extraction and processing were conducted independently by two reviewers utilizing the aforementioned databases and Mendeley Reference Manager Version 2.149.0 (which was also used to remove duplicates). The selection process has been presented using a PRISMA flowchart (Figure 2). From an initial pool of 1033 records, 140 were retrieved for secondary assessment based on a primary screening of titles and abstracts. The majority of original studies were excluded for failing to meet the inclusion criteria, specifically those unobtainable in English (*n* = 2), involving animal models (*n* = 3), being the wrong article type (*n* = 12), not examining AF (*n* = 41), or lacking psychological variables or linked lifestyle factors (*n* = 64). All exclusion criteria, as well as information on two papers identified during the update and gray literature search, are shown in Figure 2. The selection process was conducted independently by two investigators.

### 2.3. Data Extraction and Management

During data extraction, the following data points were sought: (1) sample size (trial and control group if provided); (2) maternal age; (3) trimester in which study was performed; (4) time and method of mental state evaluation; (5) time and method of amniotic fluid collection and analysis; (6) inclusion and exclusion criteria; (7) smoking status; (8) presence of other factors; and (9) reported changes in AF and other important conclusions.

### 2.4. Quality Assessment

The quality of all studies was evaluated using the Quality Assessment Tool for Observational Cohort and Cross-Sectional Studies designed by the National Institutes of Health (NIH) [31]. Articles meeting the inclusion criteria were independently evaluated for quality by two investigators. Any divergent ratings were subsequently addressed through consensus-based discussion to ensure reliability. The results of quality assessment, as well as search strategy, are available in the Supplementary Materials.

### 2.5. Data Synthesis and Limitations

Due to the methodological and clinical heterogeneity of the included studies, a narrative synthesis was performed. The primary measures used to evaluate and present the outcomes were the direction of correlations (positive, negative, or lack thereof) between maternal factors and amniotic fluid parameters, as well as the observed differences in mean biomarker concentrations between the study and control groups. Studies were divided into four groups regarding maternal factors: mental state, psychiatric treatment, smoking, and other factors.

Although the aim was to include all relevant data, the broader applicability of conclusions may be limited by publication bias and inconsistencies in how psychological stress is measured. Every effort was made in order to provide a comprehensive assessment of the study’s limitations. Nevertheless, adherence to the PRISMA 2020 standards maximizes the clarity and replicability of this study.

## 3. Results

**Figure 3:**
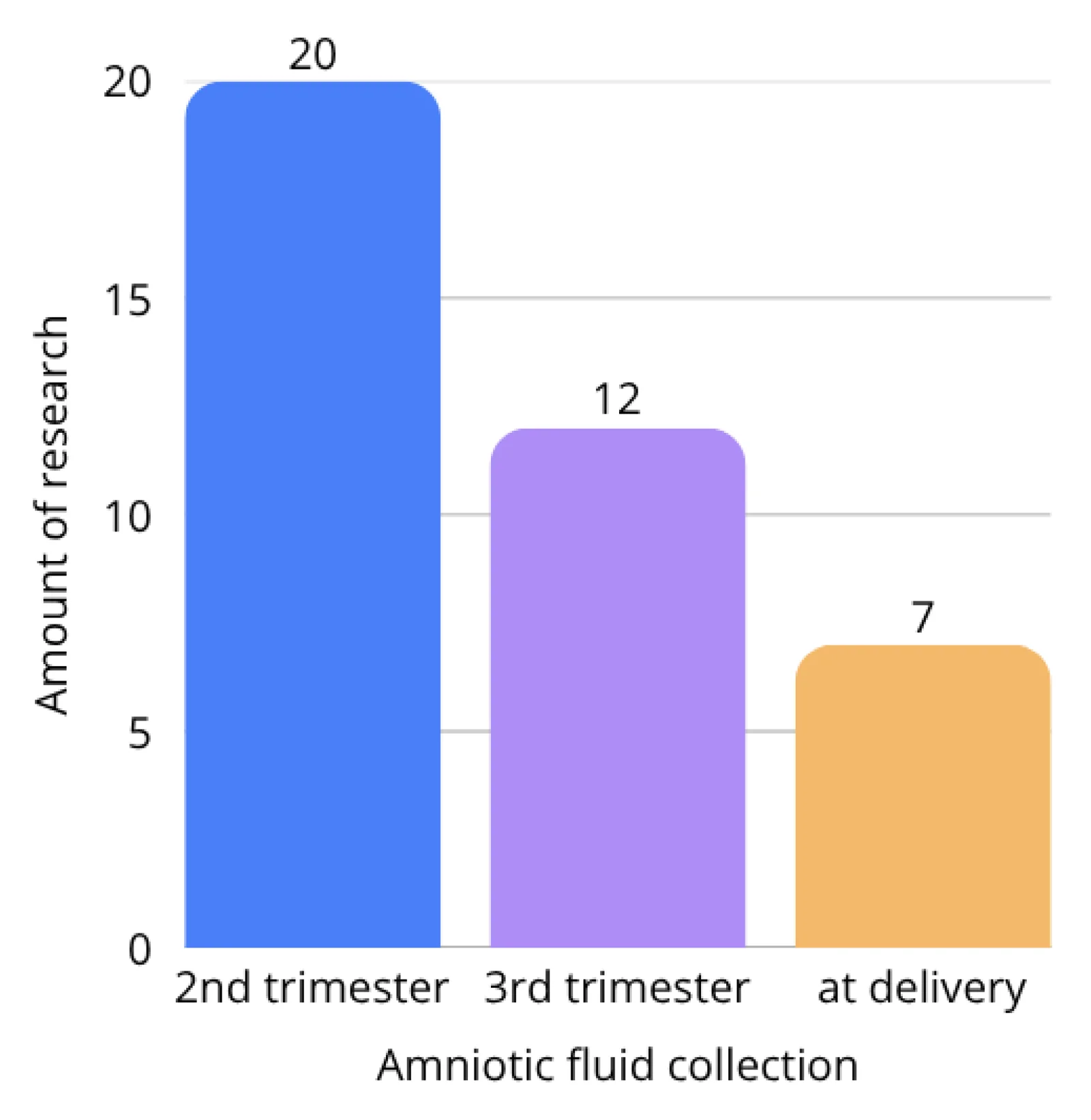
Timing of amniotic fluid collection in the included studies—studies may be included in more than one category (for at delivery: vaginal *n* = 1, cesarean section *n* = 3, both *n* = 3).

Time of sample collection is a crucial factor in results analysis (Figure 3). The majority of included studies examined amniotic fluid obtained in the second trimester (*n* = 20), a considerable number were conducted using samples collected in the third trimester (*n* = 12), while only a few researchers looked into samples obtained not by amniocentesis but collected during delivery (vaginal *n* = 1, cesarean section *n* = 3, both *n* = 3).

### 3.1. Mental State

**Figure 4:**
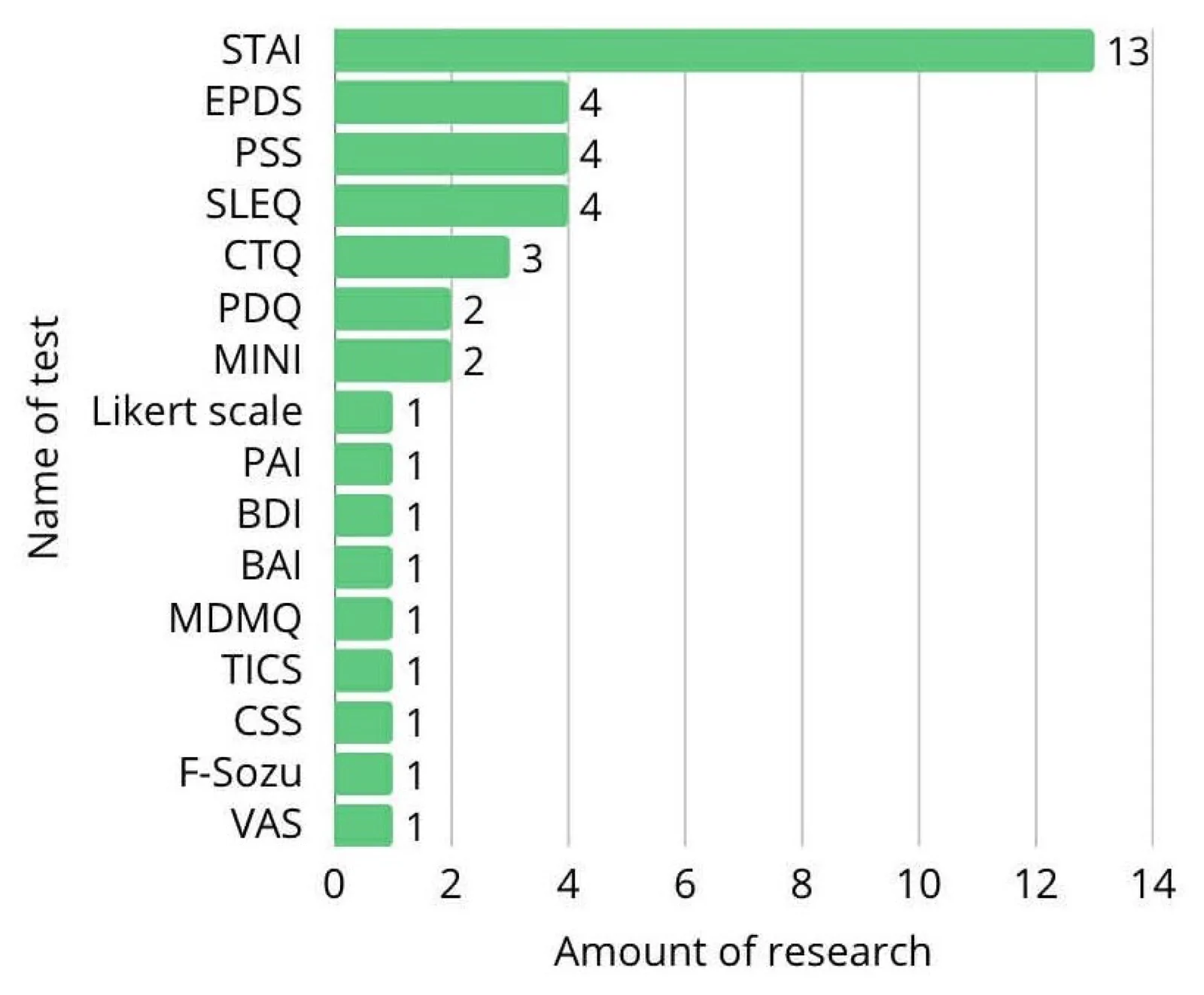
Questionnaires used to evaluate mental state in included studies; Abbreviations: BDI—Beck Depression Inventory; BAI—Beck Anxiety Inventory; CSS—Chronic Stress Scale; CTQ—Childhood Trauma Questionnaire; EPDS—Edinburgh Postnatal Depression Scale; F-Sozu—Questionnaires on Social Support; MDMQ—Multidimensional Mood State Questionnaire; MINI—Mini-International Neuropsychiatric Interview; PAI—Prenatal Attachment Inventory; PDQ—Personality Diagnostic Questionnaire; PSS—Perceived Stress Scale; SLEQ—Sleep Experience Questionnaire; STAI—State-Trait Anxiety Inventory; TICS—Trier Inventory for Chronic Stress; VAS—Visual Analog Scale.

**Figure 5:**
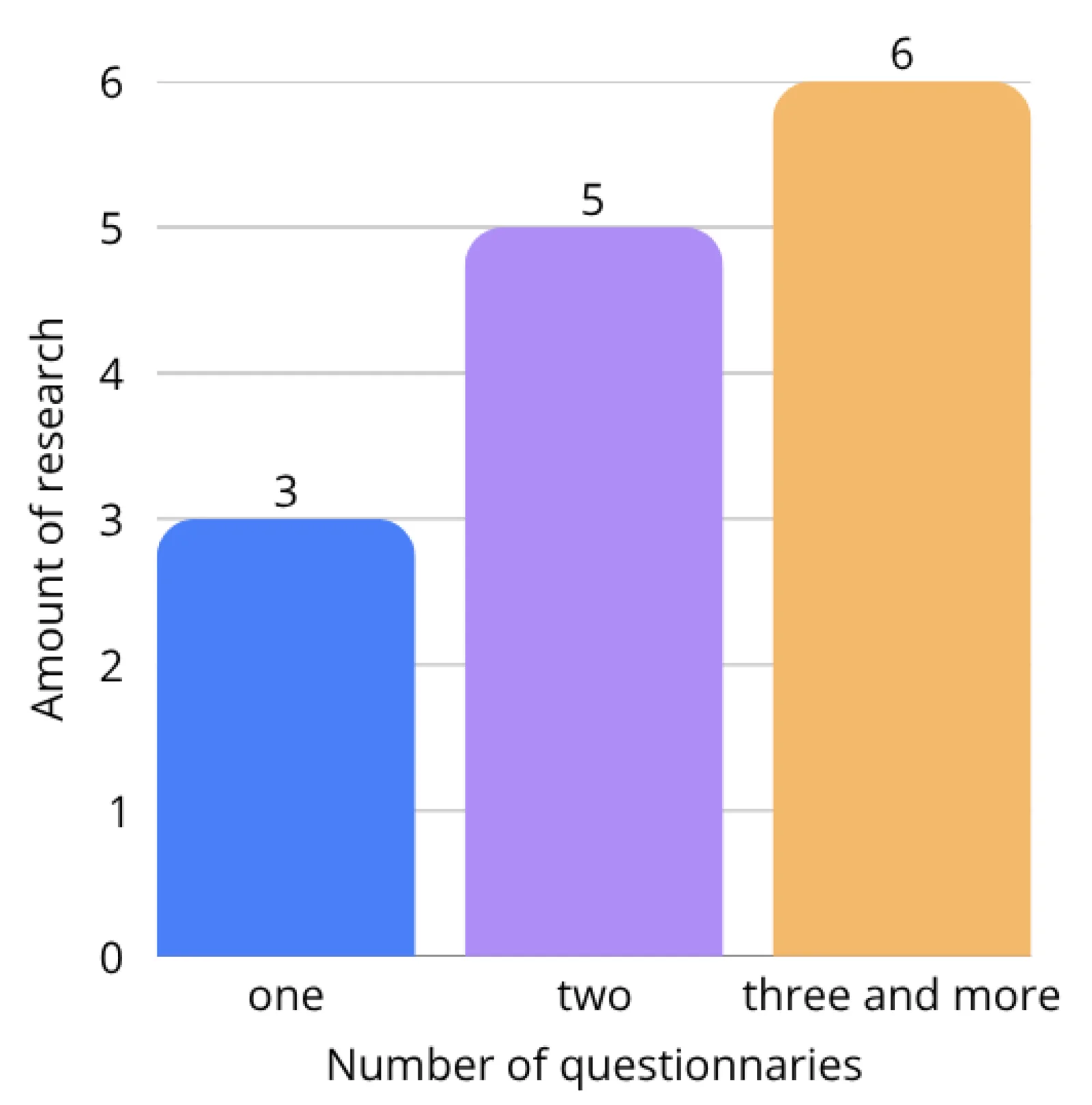
Number of tools used to evaluate mental state in studies (studies that did not specify the assessment tools *n* = 2).

A total of sixteen papers concerning the influence of maternal mental state on various components of amniotic fluid were identified (Table 1). The majority of studies (11 out of 16) determined cortisol concentrations, a key stress hormone [33]. Maternal mental state has been assessed by commonly used clinical questionnaires, including STAI, EPDS, PSS, SLEQ, CTQ, PDQ, MINI, Likert scale, PAI, BDI, BAI, MDMQ, TICS, CSS, F-Sozu and VAS (Figure 4), which had been given to mothers either pre- or postpartum. Most researchers assessed mental state prior to parturition (12 out of 16), while few studies assessed mental state after delivery (4 out of 16). Among the sixteen studies, three used a single test, six used two questionnaires, and six relied on multiple assessment tools (Figure 5). This allowed researchers to better capture various aspects of stress, including acute vs. chronic, pregnancy-specific vs. general, and state vs. trait stress. Two studies did not specify the names or numbers of questionnaires used. The collection of samples of amniotic fluid was performed either during the second trimester (7 out of 16), third trimester (2 out of 16) or both (7 out of 16), ranging from the 14th to 41st week of gestation.

According to five studies, maternal mental state was found to be independent of cortisol levels in amniotic fluid [34,35,36,37,38]. Nevertheless, four studies indicated a strong positive correlation between cortisol levels in amniotic fluid and maternal plasma cortisol levels [34,36,38,39], and two studies showed that the presence of maternal anxiety amplified this correlation [36,38].

Elevated stress levels were also linked to the shift in fetal cortisol metabolism characterized by increased 5α-reductase activity and diminished CYP3A7 activity [40]. This shift could result in fetuses being more exposed to elevated levels of active cortisol due to decreased conversion of cortisol into 6-hydroxycortisol. Excessive exposure to cortisol has been linked to impaired cognitive abilities in infants [35].

Amniotic fluid cortisol level positively correlates with amniotic fluid testosterone level [41]; however, neither this relationship nor testosterone concentration itself was found to be dependent on maternal anxiety levels [38,41,42]. Such correlations may arise from the shared nature of cortisol and testosterone as steroid derivatives [43].

Four out of sixteen papers also investigated the influence of acute stress induced by the amniocentesis procedure on amniotic fluid parameters. One study showed a negative correlation between acute anxiety and amniotic cortisol levels [44], in contrast to another study indicating that neither amniotic cortisol nor cortisone levels were associated with acute stress [45]. Another study showed a negative relationship between maternal autonomic system activation and amniotic fluid cortisol levels; however, the autonomic nervous system (ANS) parameter was found to be unrelated to maternal psychological response to amniocentesis [46]. Furthermore, acute stress had no influence on corticotropin-releasing hormone (CRH) amniotic levels, while prolonged stress was linked to elevated CRH levels in amniotic fluid [47], which is reasonable considering the time required to synthesize and release proteins such as CRH.

One study investigated the influence of maternal mental state on amniotic fluid noradrenaline levels by analyzing the correlations of both these factors with fetal activity [48]. Concentrations of catecholamines, mainly noradrenaline, in amniotic fluid were found to be possibly useful in evaluating and predicting fetal stress [49]. Elevated noradrenaline levels were linked to increased fetal activity; however, the study did not demonstrate a significant correlation between fetal behavior and maternal anxiety [48].

Two papers were identified that discussed the associations between maternal mental state, amniotic fluid brain-derived neurotrophic factor (BDNF), and amniotic cortisol levels [44,50]. BDNF is involved in crucial processes mainly within the nervous system, including growth, differentiation and survival, retrograde transport in neurons, induction of Schwann cell migration, synaptogenesis, and lymphopoiesis [51]. As BDNF levels were found to be notably higher in the amniotic fluid of fetuses with impaired growth, it can be considered a significant factor in the prediction of fetal development [51]. However, papers that were analyzed appeared to be inconsistent regarding the influence of maternal mental state on amniotic BDNF levels, as one study showed no significant correlation between those factors [50] and the other one indicated that BDNF has a positive correlation with maternal early-life adversity [44]. Both studies used the same Childhood Trauma Questionnaire (CTQ) to assess this aspect of maternal mental state, but they differed in the time taken to evaluate mental state; in the study by Deuschle et al., psychiatric assessments were completed in the week following the amniocentesis [44], whereas Lamadé et al. performed the assessments within one to seven days prior to elective cesarean section [50].

Moreover, data on the relationship between amniotic BDNF and amniotic glucocorticoid levels were also contradictory, as one paper indicates no significant correlation between these factors [44] and the other showed a positive association between these parameters [50].

Maternal childhood trauma identified by the CTQ was also negatively correlated with fetal insulin-like growth factors IGF-1 and IGF-2. IGF is a molecule that strongly correlates with fetal growth and length, and its defect has been associated with growth retardation and impaired skeletal maturation [52]. In contrast, other psychological measures, including maternal depression, anxiety, and perceived stress, as well as acute stress associated with amniocentesis, did not influence the concentrations of IGF1 and IGF2 [53].

**Table 1:** Impact of psychological factors on amniotic fluid composition.

Ref.	Year	Sample Size	Maternal Age [Years]	Pregnancy Trimesters	Time of Mental State Evaluation	Method of Evaluating Mental State	Time of Sample Collection [Week]	Method of Analysis	Inclusion Criteria	Exclusion Criteria	Results
[50]	2024	37	23–40 (31.59)	3	1–7 days prior to elective cesarean section	EPDS, PDQ, PSS, CTQ, PA and maternal social support (F-Sozu), STAI	At delivery–cesarean section 37.14–41 (38.80)	BDNF-ELISA; LCMS (cortisol and cortisone)	Ability to consent, maternal age ≥18 years and <51 years, ability to speak German	Drug or alcohol addiction, a substitution therapy, severe infections (such as hepatitis and HIV), amniotic fluid samples contaminated with blood	No correlation between maternal psychological factors and amniotic BDNF levels. Positive correlation between amniotic BDNF levels and amniotic cortisol and cortisol levels
[53]	2021	79	22–45 (36.3)	2	NS	Likert scale 0 to 10; STAI; MINI, PDQ (pregnancy-specific stress), CTQ	15.9 ± 0.9	Blocked RIA (IGF-binding protein)	German-speakers, ability to consent, age above 18 years	Blood contamination of amniotic fluid, structural abnormalities of fetus	Negative correlation between maternal child trauma and amniotic IGF-1, IGF-2 levels. Negative correlation between maternal BMI and amniotic IGF-1, IGF-2 levels. No correlation between acute stress and amniotic IGF-1, IGF-2 levels. No correlation between maternal anxiety, depression, perceived stress and amniotic IGF-1, IGF-2 levels.
[44]	2018	79	22–45 (36.3)	2	In the week after the amniocentesis	MINI, EPDS, STAI, PSS, CTQ, Likert scale	14–19 (15.9)	BDNF-ELISA	Singleton pregnancies, structurally normal fetus, appropriate fetal growth	Aneuploidy, fetal structural abnormalities, contamination of amniotic fluid sample with blood, taking medications other than thyroid hormones and vitamin supplementation	Positive correlation between maternal early-life adversity and amniotic BDNF levels No correlation between BDNF levels and glucocorticoid levels or HSD2 activity. Negative correlation between acute stress and amniotic cortisol levels. No correlation between acute stress and cortisone levels
[47]	2017	34	18–45	2	40 and 10 min before and 20 min after amniocentesis; after receiving normal amniocentesis test result (TICS)	STAI, CSS, TICS	15–18 (15.9)	RIA	Singleton intrauterine pregnancy undergoing amniocentesis for karyotyping	Artificial insemination, known maternal and fetal medical complications, suspected or known fetal growth restriction, ultrasound confirmed fetal structural anomalies, maternal psychiatric disorder, smoking or alcohol consumption of more than one glass of wine or beer per week, medication use (e.g., glucocorticoids, psychotropic drugs, diuretics, antihypertensives, and vasodilators), and food and/or protein restrictions (e.g., vegetarian or vegan diet)	No correlation between acute stress and amniotic fluid peptides, positive correlation between chronic stress and amniotic CRH
[40]	2015	AGA *n* = 399 SGA *n* = 343	AGA 30–36 SGA 29–37	3	Between 24 and 40 weeks gestation	PSS, STAI	At delivery–Cesarean section or speculum examination during vaginal delivery AGA 38.9–40.7 SGA 36.6–39.5	LCMS	Low-risk pregnancies in third-trimester routine pregnancy care	Maternal psychiatric disorders requiring medication, confirmed chromosomal or fetal anomaly, incomplete follow-up	Positive correlation between maternal anxiety and increased 5α-reductase activity and diminished CYP3A7 activity in fetuses
[46]	2015	34	27–45 (37.38)	2	40 and 20 min before and 20 min after amniocentesis	Multidimensional Mood State Questionnaire	15–18 (15.96)	HPLC-UV	Healthy pregnant women, singleton intrauterine pregnancy	Artificial insemination, current mental disorders, medication, smoking, obesity	No correlation between maternal stress and amniotic fluid parameters or maternal ANS activity. The findings suggest that the maternal ANS plays a critical role in regulating the fetoplacental barrier’s response to stress
[45]	2013	34	37.38	2	40, 10 and 1 min before and 20 min after amniocentesis	STAI, VAS	(15.96)	LCMS/MS	Referral for second-trimester amniocentesis	Artificial insemination, multiple gestation, known maternal or fetal medical complications, maternal psychiatric disorders, protein-restricted diet, alcohol intake of more than one glass of wine or beer per week, current use of psychotropic substances or medication	Positive correlation between acute stress and salivary cortisol and cortisone Positive correlation between salivary cortisol and amniotic cortisone
[34]	2012	125	25–14 (36.68)	2–3	prenatal (STAI) postnatal (STAI, SLEQ, EPDS)	STAI, SLEQ, EPDS	15–37 (90% 15–20)	RIA	Full-term pregnancy, healthy and singleton infant	Clinical findings, prematurity, non-routine amniocentesis or unknown birth outcome	No correlation between maternal anxiety and cortisol Positive correlation between maternal plasma cortisol and amniotic cortisol
[37]	2012	158	28–45 (37.6)	2	PSS (2 weeks before amniocentesis), STAI (15 min prior to amniocentesis)	PSS, STAI	15–18 (16.41)	RIA	Healthy singleton pregnancy (abnormalities of the thyroid gland, if well-treated, were allowed)	Twin pregnancy, diabetes, chronic use of steroid ointment, intake of hormonal medication, sex chromosome deviation, terminated pregnancy, lack of postnatal data, general anesthesia during pregnancy	No correlation between maternal anxiety and both amniotic and maternal plasma cortisol
[35]	2010	125	25–45 (36.62)	2–3	prenatal (STAI) postnatal (EPDS)	STAI, EPDS, SLEQ	15–37 (17.30)	RIA	Not meeting any exclusion criteria	Clinical findings, prematurity, non-routine amniocentesis, or unknown birth outcome	No correlation between maternal anxiety and amniotic cortisol
[42]	2010	107	28–45 (36.95)	2–3	postnatal visit (state anxiety additionally on prenatal/amniocentesis visit)	SLEQ, STAI	15–32 (17.19)	RIA, LCMS	English-speakers, full-term (≥37 weeks), healthy, singleton infants, known birth outcomes	Known abnormalities, incomplete data on birth outcome, non-routine amniocentesis	No correlation between maternal anxiety and amniotic or prenatal testosterone levels
[36]	2008	262	18–47 (35.3)	2–3	NS	STAI	15–37	RIA	Singleton pregnancies, structurally normal fetus, appropriate fetal growth on ultrasound	Fetal structural abnormalities, samples visually contaminated with blood, samples subsequently associated with fetal aneuploidy or fetal death	No correlation between maternal anxiety and amniotic cortisol Correlation between maternal and amniotic cortisol in more anxious women
[38]	2008	317	NS	2–3	STAI 15 min prior to amniocentesis	STAI-S/T + SLEQ (postnatally)	15–37 (17)	RIA	Singleton pregnancies, structurally normal fetus, appropriate fetal growth on ultrasound	Fetal structural abnormalities, samples visually contaminated with blood, samples subsequently associated with fetal aneuploidy or fetal death	Positive correlation between maternal plasma cortisol and amniotic cortisol was amplified by maternal anxiety. Maternal anxiety itself did not show any correlation with amniotic cortisol or testosterone.
[39]	2007	267	18–47	2–3	NS	NS	15–37	RIA	Informed, written consent to participate in the study	History of clinical depression, taking steroidal medication for chronic disease	Positive correlation between amniotic fluid cortisol with maternal plasma levels
[41]	2007	264	18–47	2–3	NS	NS	15–37	RIA	Singleton pregnancies, structurally normal fetus, appropriate fetal growth on ultrasound	Fetal structural abnormalities on preprocedural scan, procedures performed for nonroutine indications, samples visually contaminated with blood and those subsequently associated with fetal aneuploidy or fetal death	Positive correlation between amniotic fluid testosterone levels and amniotic cortisol levels No correlation with maternal anxiety.
[48]	2003	trial: 10 control: 10	trial: 33–43 (38.1 ± 4.9) control: 25–32 (28.9 ± 3.9)	2	trial: given on the day of amniocentesis and collected 2 days later control: given the questionnaire and booked for a fetal observation scan 2 days later.	STAI	trial: 40–41 (40.1 ± 0.7) control: 38–40 (39.1 ± 1.5)	RIA (T4, T3, TSH, cortisol and ACTH), HPLC (catecholamines)	NS	NS	No correlation between maternal anxiety and fetal behavior Positive correlation between fetal activity and lowered maternal glucose levels and elevated levels of noradrenaline in the amniotic fluid

Values in parentheses represent mean maternal or gestational age. Abbreviations: ACTH—Adrenocorticotropic Hormone; AGA—Appropriate for Gestational Age; ANS—Autonomic Nervous System; BDNF—Brain-Derived Neurotrophic Factor; CRH—Corticotropin-Releasing Hormone; CSS—Chronic Stress Scale; CTQ—Childhood Trauma Questionnaire; ELISA—Enzyme-Linked Immunosorbent Assay; EPDS—Edinburgh Postnatal Depression Scale; F-Sozu—Questionnaires on Social Support; HIV—Human Immunodeficiency Virus; HPLC-UV—High-Performance Liquid Chromatography with UV-Vis Detection; HSD2—11beta Hydroxysteroid Dehydrogenase 2; IGF—Insulin-like Growth Factor; LCMS—Liquid Chromatography–Mass Spectrometry; MINI—Mini-International Neuropsychiatric Interview; NS—Not Specified; PA—Prenatal attachment to the offspring; PDQ—Personality Diagnostic Questionnaire; PSS—Perceived Stress Scale; RIA—Radioimmunoassay; SGA—Small for Gestational Age; SLEQ—Sleep Experience Questionnaire; STAI—State-Trait Anxiety Inventory; T3—triiodothyronine; T4—tetraiodothyronine; TICS—Trier Inventory for Chronic Stress; TSH—Thyroid-Stimulating Hormone; VAS—Visual Analog Scale.

### 3.2. Psychiatric Diagnosis and Treatment

In some cases, psychiatric disorders may require administration of medications, for instance, selective serotonin reuptake inhibitors (SSRIs). These drugs exhibit a high propensity for placental passage. According to recent studies, antidepressant drugs and their metabolites are present in 86.8% of umbilical cord samples, with the lowest ratios for sertraline and paroxetine and the highest ratios for citalopram and fluoxetine [54]. Furthermore, SSRI use during pregnancy has shown a potential association with severe adverse effects in infants, including necrotizing enterocolitis and seizures [55].

Three studies were identified concerning the influence of selective serotonin reuptake inhibitor administration during the third trimester of pregnancy on the levels of S100B and activin A in amniotic fluid (Table 2). In all cases, the treatment was prescribed by a psychiatrist due to diagnoses of severe mental disorders including major depression, panic disorders, a combination of both, generalized anxiety disorders, obsessive–compulsive disorder or bulimia. All studies comprised women receiving SSRIs for at least six months; however, two of these included only women treated by paroxetine [56,57], while one also included women treated with fluvoxamine, fluoxetine, and sertraline (aside from paroxetine) [58]. Two studies investigated S100B levels [56,57] and the remaining one assessed activin A levels [58]. Elevated concentrations of both can indicate neuronal damage and have been correlated with the occurrence of cerebral bleeding in infants [56,58]. In all considered articles, the concentrations of the markers studied in amniotic fluid were significantly higher in the SSRI groups than in controls. Moreover, two studies associated the highest concentrations of S100B with the presence of neurological symptoms in infants, including hypotonia and seizures. Hypotonia (10/50) and seizures (4/50) were reported in the paroxetine-exposed group. No cerebral hemorrhage was detected on neonatal ultrasound. These short-term findings should not be equated with demonstrated structural neuronal injury or persistent neurodevelopmental impairment [56,57].

Some psychiatric conditions, including generalized anxiety disorder and a high prevalence of major depressive disorder, were found to be associated with oligohydramnios. In the majority of cases, the psychiatric diagnoses preceded the detection of this condition, suggesting that psychiatric disorders may be a predisposing factor for the occurrence of oligohydramnios [59].

### 3.3. Smoking

Poor maternal mental health and stress are often associated with the adoption of harmful behaviors, such as cigarette smoking [60]. Three papers were identified addressing maternal smoking during pregnancy (Table 3). All studies clearly demonstrated that maternal smoking during pregnancy has a significant impact on the properties of amniotic fluid. However, the groups of pregnant women included in these studies differed in smoking status and level of nicotine exposure. Another limitation that complicates tracking the relationship between smoking during pregnancy and its impact on amniotic fluid composition is the fact that only one of the studies compared the results with maternal cotinine levels in the mother’s blood [61]. The others relied on the mother’s report of the number of cigarettes smoked [60], or even solely on self-reported smoking without providing specific numerical values [62], which may have reduced the reliability of the results.

Cigarette smoke is both a source and an initiator of increased reactive oxygen species production in the body, contributing to enhanced oxidative stress [63]. One article suggests the presence of antioxidant substances and fetoplacental mechanisms that inhibit low-density lipoprotein (LDL) oxidation during tobacco exposure [60]. These conclusions were based on the observation that amniotic fluid from smoking mothers lengthened LDL oxidation lag phase. This is the phase preceding lipid peroxidation, in which antioxidants participate and partially prevent LDL oxidation. It reflects the antioxidant status of membranes and their resistance to oxidation—a longer lag phase indicates a higher concentration of antioxidants [64].

Nicotine exposure is also associated with alterations in other metabolic pathways in amniotic fluid, involving aspartate and asparagine, pyrimidine, urea/amino group, arginine and proline and xenobiotic metabolisms [61]. The changes include decreased levels of N1,N12-diacetylspermine, acetylspermidine, and N1-acetylspermine; increased concentrations of L-arginine and adenosine monophosphate; and reduced levels of proline, thymidine, and cytosine. Another study revealed decreased levels of adiponectin in smokers’ amniotic fluid while both insulin and pregnancy-associated plasma protein A were increased, which suggests that tobacco smoke may disrupt fetal enzymatic activity [62]. The presented results illustrate the complexity and multidimensionality of the changes induced by exposure to tobacco smoke. Alterations in enzymatic and hormonal pathways, and consequently in metabolic processes, support the plausible framework of metabolic programming during the initial stages of development.

### 3.4. Metabolic Stressors

While this review primarily focuses on maternal psychological state, the composition of amniotic fluid has been studied in relation to broader metabolic and inflammatory stressors, such as diabetes (including types I, II or gestational) [65,66], cytomegalovirus infection [67], and obesity [68,69,70]. Rather than acting primarily as psychological exposures, these conditions are best understood as potent physiological stressors that converge on common biological pathways with psychological distress. Specifically, they alter the maternal–placental–fetal triad through shared mechanisms, including elevated oxidative stress, systemic inflammation, altered placental signaling, and dysregulated fetal metabolic regulation (Table 4). Although these metabolic and inflammatory conditions frequently precipitate or coexist with deteriorating maternal mental health [71,72,73,74], their fundamental impact on the intrauterine environment is driven by these overlapping inflammatory and metabolic cascades.

Although chronic hyperglycemia and impaired antioxidant capacity in patients with diabetes are associated with enhanced oxidative stress [75,76], neither study showed a link between this metabolic condition in pregnant women and significant changes in the composition of amniotic fluid. However, one of the studies [65] reported elevated levels of growth-differentiation factor 15, produced in response to oxidative stress in preeclampsia and diabetic preeclampsia. This can be explained by the fact that this pregnancy complication is associated with placental oxidative stress [77,78].

Obesity, although associated with stress at the metabolic level, may also result from the adoption of unhealthy dietary habits by pregnant women. There are studies supporting the hypothesis that among pregnant women, those suffering from depression are more likely to develop obesity [79,80]. Articles examining pregnant women whose body mass index (BMI) indicated obesity demonstrate that this metabolic condition is associated with significantly different regulation of 205 genes, including an increase in the expression of the apolipoprotein D gene, reduced signaling in mitochondrial apoptotic pathways, and activation of estrogen receptors, FBJ murine osteosarcoma viral oncogene homolog, and signal transducer and activator of transcription 3 [68].

Changes were also observed in the levels of phosphate and uric acid in amniotic fluid, which were found to be elevated [69]. The authors point out the potential negative clinical implications for the offspring resulting from these alterations.

Only one study examined the impact of oxidative stress induced by an infectious agent, in this case cytomegalovirus (CMV) [67]. Exposure to early-life stress is a known risk factor for psychiatric disorders, with immune dysregulation that weakens adaptive immunity as a potential connection. This vulnerability may contribute to infection or reactivation of herpesviruses, such as CMV. While once considered relatively harmless, CMV is now linked to serious long-term consequences, including depression, neurodegeneration, and a shorter lifespan [72]. Pregnant patients with an active infection by this virus showed decreased concentrations of 3-nitrotyrosine in the amniotic fluid, which is a marker of oxidative damage [81]. This provides further evidence for the existence of fetal antioxidant defense mechanisms.

## 4. Discussion

This review synthesizes current knowledge regarding the influence of maternal mental state, psychiatric treatment, and environmental factors on the biochemical composition of amniotic fluid. Mental state was the most extensively studied area. Pregnant women might be exposed to elevated levels of stress associated with societal pressures, obstetric complications, or even certain diagnostic procedures. One such procedure is amniocentesis, which has been identified as a trigger for acute psychological stress [82].

Amniocentesis serves as a vital diagnostic tool but is also a significant acute psychological stressor for pregnant women, reliably inducing high levels of state anxiety and elevating maternal plasma cortisol levels [83]. This procedure-induced distress complicates the use of amniotic fluid in prenatal stress research, as the acute anxiety directly alters the fluid’s biochemical composition [84,85]. Specifically, the acute maternal autonomic response to the procedure may trigger protective mechanisms, such as the upregulation of the placental 11β-Hydroxysteroid Dehydrogenase 2 (11β-HSD2) enzyme, which protects the fetus by converting active cortisol into inactive cortisone [46]. Conversely, markers of chronic maternal stress are associated with an impaired placental barrier and higher concentrations of active cortisol in the fetal environment. Furthermore, elevated amniotic cortisol during the procedure is positively correlated with increased testosterone levels across both sexes [38]. Researchers utilizing amniotic fluid to study fetal programming must carefully account for these acute, procedure-driven fluctuations, as the anticipation and physical intervention of amniocentesis actively modify the maternal–fetal stress-related dynamic [82,84,85].

It is worth noting that the method of amniotic fluid collection for the purposes of these studies differed between articles. Most women underwent standard amniocentesis, while in seven studies the fluid was collected during delivery—either during cesarean section or via a vaginal speculum. The latter approach allowed some women to avoid the potential stress associated with amniocentesis, although delivery introduces a different physiological context. Only one study [47] directly acknowledges anxiety linked with waiting for amniocentesis results, but in cases where mental state was assessed weeks after amniocentesis or postnatally, it is highly probable that the mother had also received results.

To address the robustness of the conclusions, it is crucial to re-examine the heterogeneous results across the studies included through the lens of this acute stress confounder. For instance, studies collecting amniotic fluid during second-trimester amniocentesis are inherently measuring a biomarker profile that may be transiently altered by immediate procedural anxiety. This acute spike in maternal sympathetic activity and cortisol might mask or distort the underlying baseline associations between chronic maternal psychological distress and amniotic biomarkers. In contrast, studies collecting fluid at the time of elective cesarean section or vaginal delivery capture a different physiological state, albeit one affected by the physical stress of labor or pre-surgical apprehension. Therefore, the lack of a uniform association between maternal anxiety and amniotic cortisol, observed in several of the reviewed studies, may be largely attributable to this confounding overlay of procedural stress rather than a true absence of biological correlation. Consequently, when comparing literature, the method and timing of fluid collection must be weighed as a critical biological modifier, not merely a methodological footnote.

Many authors have attempted to investigate the relationship between questionnaire-based assessments of maternal mental health and concentrations of steroid hormones in the blood of pregnant women. Elevated amniotic cortisol and testosterone levels, which are interrelated, were found to be related to decreased body weight in infants [37,86], which may be subsequently associated with difficult infant temperament [87,88]. Furthermore, some research indicates that intrauterine exposure to excessive testosterone is linked to the development of autistic traits, such as impaired social skills, language development, empathy, systemizing, and visual analytic skills [89].

However, none of the studies identified in the review demonstrated a clear trend or significant association between the psychological state of pregnant women and circulating cortisol levels. It is also necessary to consider other factors that may have confounded the questionnaire results and their association with cortisol levels. In five studies, the questionnaires were administered after delivery, in which women were asked to recall their emotions related to the course of pregnancy (Figure 5). Some participants may have experienced postpartum depression, which could have influenced their assessment of their actual well-being during pregnancy [90,91]. In addition, there were discrepancies in the types of stress assessed (chronic and acute) and the types of questionnaires used across different studies (Figure 4). Another key factor influencing the results is the presence of 11β-HSD2, produced and released into the maternal bloodstream by the placenta [92]. Its placental expression increases throughout pregnancy, and this mechanism is believed to protect the fetus from excessive maternal glucocorticoids [93]. As a result, the presence of 11β-HSD2 in the blood could interfere with accurate monitoring of this hormone [92].

When interpreting these endocrine responses, it is crucial to consider that significant inter-individual variation in placental 11β-HSD2 expression fundamentally modulates the relationship between maternal stress and amniotic cortisol levels. A primary mechanism driving this variability is epigenetic regulation, specifically the DNA methylation of the *HSD11B2* gene promoter, which may influence the degree of transcriptional repression and subsequent enzyme activity [94,95]. For instance, chronic maternal distress and anxiety can induce hypermethylation of this promoter, thereby weakening the enzymatic barrier and increasing the transfer of active cortisol into the fetal compartment [96]. Furthermore, these inter-individual differences are heavily influenced by fetal sex dimorphism; research indicates that placental *HSD11B2* expression and methylation patterns in response to maternal psychological challenges frequently differ between male and female fetuses [97]. However, the scientific literature presents notable discrepancies regarding the exact impact of maternal stress on 11β-HSD2 function, with numerous studies reporting increased, decreased, or entirely unchanged enzyme activity [98]. These conflicting results are hypothesized to arise from methodological differences in stress assessment, varying processing times for placental samples, failure to account for confounders such as maternal body mass index or smoking, and the specific timing and intensity of the stressor [94]. To reconcile these inconsistencies, a disrupted adaptive framework has been proposed, suggesting that while acute maternal stress may initially upregulate 11β-HSD2 expression as a compensatory protective mechanism, prolonged chronic stress exhausts this capacity [99]. This chronic exhaustion ultimately leads to the epigenetic downregulation of the enzyme, leaving the fetus vulnerable to elevated maternal cortisol and subsequent developmental programming alterations. Additionally, research among mothers with depression showed that antidepressant treatment alone is unlikely to protect against this enzymatic alteration [100]. Since studies regarding 11β-HSD2 often rely on animal models or small sample sizes of maternal–fetal dyads, this area requires further research to establish definitive clinical conclusions [94,98].

Moreover, one study showed no significant correlation between maternal salivary cortisol levels and amniotic fluid cortisol levels [47] despite clear associations between maternal plasma and amniotic fluid cortisol as well as between plasma and salivary cortisol levels [101]. This might support the hypothesis of altered metabolism of cortisol in pregnant women. Therefore, it may be worth considering the investigation of alternative factors that could better correlate with the psychological state of pregnant women, such as pro-inflammatory cytokines interleukin-6 (IL-6) and tumor necrosis factor alpha (TNF-α), whose blood concentration increases in chronic psychological stress [102]. This is also supported by clinical evidence demonstrating that pro-inflammatory factors, including IL-6 and CRP, are strongly associated with anxiety severity and may decrease following SSRI treatment, suggesting a relationship between psychological state and inflammatory signaling [103].

In some cases, maternal psychological distress may require psychiatric medication. Although depression during pregnancy is not as widely discussed as postpartum depression (PPD), it may represent a similar or even more prevalent problem than PPD [104]. Research regarding the impact of SSRIs on breast milk composition has shown that these drugs significantly alter its biochemical makeup, primarily concentrations of IL-10, IL-6, allopregnanolone, vitamins, myeloperoxidase, lipid peroxidase, and lipid profile [105]. This suggests a potential link between this factor and the composition of amniotic fluid. According to the most recent analyses, at least one in ten women is exposed to a psychotropic medication during the perinatal period [106]. As mentioned earlier, SSRIs, which were the subject of examination in the majority of studies, readily cross into the amniotic fluid and are associated with an elevated risk of fetal neurological effects.

Guidelines for treating depression during pregnancy emphasize a personalized approach that balances clinical efficacy with safety. Recommendations vary depending on the country of origin or organization that establishes guidelines. For mild to moderate depression, psychotherapy is generally recommended as the preferred initial treatment, whereas antidepressants are typically reserved for more severe cases [107]. A notable exception is the American College of Obstetricians and Gynecologists, which favors pharmacological treatment regardless of symptom severity [108]. For patients already on medication, several guidelines advise continuing the current drug regimen in order to maintain psychological stability, though Danish recommendations suggest switching medications if the patient is using drugs with less favorable profiles, such as paroxetine or fluoxetine [107,108]. Any decision to continue, taper, or switch medications should be based on a thorough discussion between the physician and the patient regarding the potential risks and benefits to ensure informed consent [107,109].

While this review highlights an association between SSRI exposure and elevated neuronal injury markers (S100B, activin A) in amniotic fluid, interpreting these findings requires significant caution. First, it is crucial to clearly distinguish between biomarker elevation, association with SSRI exposure, transient neonatal neurological findings, and demonstrated structural neuronal injury [110]. Furthermore, one must differentiate between potential drug-induced effects and the biological impact of the underlying maternal psychiatric illness. The severity of maternal depression or anxiety acts as a major confounder; therefore, elevated biomarkers may partially reflect the pathophysiology of the untreated or severe psychiatric condition itself, rather than isolated drug toxicity. Furthermore, the current literature analyzing amniotic fluid biomarkers does not sufficiently differentiate risk profiles among individual SSRIs. The available data predominantly focus on paroxetine, which has been shown to exert dose-dependent effects [57]. However, there is a critical lack of studies providing head-to-head comparisons between paroxetine and other commonly prescribed SSRIs, such as sertraline, citalopram, or fluoxetine. Distinguishing these risk profiles is clinically vital, as medications like sertraline are often considered first-line treatments during pregnancy due to their more favorable safety profiles, whereas paroxetine has been historically associated with a higher risk of adverse neonatal outcomes. Future research must stratify risk by specific SSRI type, exact dosage, and the severity of the maternal psychiatric condition to properly guide safe pharmacological interventions during pregnancy [111].

Maladaptive behaviors, such as smoking and unhealthy eating habits, remain widespread problems among pregnant women. Recent studies show that 52.9% of women who previously smoked cigarettes daily continued this habit after becoming pregnant [112]. However, it turns out that even passive exposure to tobacco smoke can disrupt the composition of amniotic fluid, thereby adversely affecting the fetus [61]. Both nicotine and cotinine—a metabolite of nicotine breakdown—present in the mother’s blood, cross the placenta exceptionally quickly. Moreover, cotinine tends to accumulate on the fetal side, suggesting that the fetus is exposed to higher concentrations of cotinine than the mother [113]. High concentrations of cotinine in amniotic fluid are associated with pregnancy complications, most commonly including low birth weight, an increased risk of miscarriage, and the development of respiratory distress syndrome [113,114]. Nicotine also reaches higher concentrations in placental tissue compared with maternal plasma [115]. Newborns exposed to this substance, in addition to the complications mentioned above, were more likely to die from sudden infant death syndrome [116].

While this scoping review highlights significant alterations in mid-gestation amniotic biomarkers, the direct causal links between these specific fluid changes and long-term offspring outcomes should currently be viewed as a plausible framework rather than a proven developmental trajectory. For instance, the dysregulation of mid-gestation amniotic cortisol, BDNF, and neuronal injury markers implies an altered neurodevelopmental milieu. Theoretically, this disturbed intrauterine environment may increase fetal susceptibility to later neurodevelopmental and psychiatric disorders, which has been proposed as a foundational mechanism for the increased risk of neurodevelopmental disorders, such as ADHD and schizophrenia, observed in prenatally stressed or nicotine-exposed offspring [117,118,119]. Similarly, metabolic sequelae, including low birth weight and impaired skeletal maturation, can be mechanistically linked to the depletion of anabolic factors like IGF-1 and IGF-2, alongside altered adiponectin and increased oxidative stress markers in the amniotic fluid [53,120]. However, it is imperative to acknowledge that the majority of the reviewed studies rely on a single mid-gestation amniotic fluid measurement without extended longitudinal follow-up. Therefore, while these biomarker shifts provide a compelling snapshot of a perturbed intrauterine environment, long-term prospective cohort studies tracking infants into childhood and adolescence are required to evaluate the proposed link between mid-gestation amniotic profiling and the subsequent development of these neurodevelopmental and metabolic outcomes [117].

Many of the investigated factors, such as smoking and obesity, may both result from poor maternal mental health and function as contributors to metabolic stress affecting mother and fetus. These factors alter the fetal environment at enzymatic [62], hormonal [60,61,62] and genetic [68] levels, which may be linked to pregnancy complications and impaired offspring development. This conclusion supports the concept of metabolic programming, which is based on the premise that early-life exposures, including maternal body composition and maternal dietary intake, can permanently modify organ structure and physiological function in the developing child [117]. Metabolomic studies have provided further insight into the molecular mechanisms underlying these alterations, offering a plausible framework for fetal metabolic programming. Although metabolomic studies directly examining maternal mental health are scarce, other factors such as nutrition, inflammation, and nicotine exposure have been shown to establish altered set points for key metabolic pathways [11]. Maternal diet is suspected to influence energy, amino acid, and choline metabolism, as well as the urea cycle and fumarate generation during purine biosynthesis [121]. Similarly, maternal smoking correlates with the deregulation of fetal aspartic acid and nucleic acid metabolism [61]. Furthermore, maternal stress can directly alter the set points for fetal nutrient availability by shifting the expression of placental glucose transporters (such as decreasing GLUT1 and increasing GLUT3 and GLUT4) and downregulating nutrient-sensitive proteins like O-GlcNAc transferase [94]. Crucially, the stress-induced reduction of OGT not only disrupts nutrient transport but also alters downstream placental transcriptional regulation. This mechanism appears to be sexually dimorphic; because male placentas exhibit lower baseline OGT expression, they may be more vulnerable to falling below a critical protective threshold under stress. Importantly, this sex-specific vulnerability and its association with maternal stress have been corroborated in human placental tissue [122]. Research on the amniotic fluid transcriptome also reveals that metabolic stressors, such as maternal obesity, are linked to the misregulation of genes and pathways involved in fetal neurodevelopment, notably showing a pronounced upregulation of APOD. These findings suggest that maternal obesity may create a pro-estrogenic and pro-inflammatory intrauterine environment [68]. Ultimately, these transcriptomic and metabolic shifts likely reflect underlying epigenetic modifications. For example, prenatal stress and altered placental signaling can induce targeted DNA methylation changes, such as the hypermethylation of the fetal glucocorticoid receptor (Nr3c1) and placental 11β-HSD2 promoters, providing a direct biological mechanism through which temporary maternal exposures permanently reprogram the offspring’s neuroendocrine and metabolic responses [94,123].

## 5. Conclusions

This scoping review demonstrates that maternal psychological state and related lifestyle factors are associated with measurable alterations in the biochemical composition of amniotic fluid. Evidence suggests that chronic stress, psychiatric treatment, and smoking may influence hormonal, metabolic, and neuronal markers within the intrauterine environment, including the amniotic fluid, which appears to contribute to alterations in cortisol, corticotropin-releasing hormone, and neuronal injury markers such as S100B and activin A.

These findings support the concept of fetal metabolic programming, highlighting the role of amniotic fluid as a dynamic component and potential mediator between maternal condition and fetal development. However, the interpretation of these results is limited by methodological heterogeneity, variability in stress assessment, and challenges in amniotic fluid sampling. The amniocentesis procedure itself may act as an acute stressor, potentially confounding the assessment of chronic maternal stress and introducing a procedure-induced bias. Furthermore, due to the nature of the procedure, which usually is not repeated in a single mother, it is impossible to consistently track changes occurring during pregnancy—all conclusions have been made from a single aliquot obtained from an individual mother during pregnancy.

Ultimately, the lack of a straightforward linear association between maternal anxiety and amniotic cortisol concentrations, despite more consistent associations with maternal plasma cortisol, should not be interpreted as evidence for the absence of a biological effect. Instead, these findings likely reflect the complex and dynamic regulation of fetal glucocorticoid exposure, which integrates maternal cortisol availability, placental glucocorticoid-buffering capacity, gestational timing, and acute stress related to the sampling procedure.

In questionnaire-based studies, there is also a risk of reporting bias. In the case of articles selected for this review, it may be present in the form of self-reporting bias (mainly maternal smoking status and social pressure perceived by mothers) and recall bias. The latter, present when data were collected postpartum, may have been influenced not only by a lack of accurate recall of their mental state but also by conditions such as postpartum depression or “maternity blues”.

However, a clear distinction must be made between these biological findings and their clinical application. Current evidence does not establish the routine clinical or predictive use of amniotic fluid biomarkers. Further research is required to better characterize the mechanisms underlying these relationships and to evaluate the clinical relevance of amniotic biomarkers in predicting offspring health. Such extended longitudinal tracking, from maternal exposure via amniotic/placental response to long-term outcome, is essential to definitively determine whether these amniotic biomarkers function as causal intermediates, compensatory physiological responses, direct exposure markers, or simply correlates of maternal distress. From a public health perspective, understanding the impact of maternal psychological and lifestyle factors on the intrauterine environment is essential for identifying early determinants of fetal development and optimizing long-term health outcomes. The presented findings support the claim that psychological support should be included in holistic care for mothers during pregnancy.

## Figures and Tables

**Table 2:** Impact of psychiatric diagnosis and treatment on amniotic fluid composition.

Ref.	Year	Sample Size	Maternal Age [Years]	Pregnancy Trimesters	Method of Evaluating Mental State	Time of Sample Collection [Week]	Method of Analysis	Inclusion Criteria	Exclusion Criteria	Results
[57]	2020	trial: 50 control: 150	trial: 31 ± 4 control: 29 ± 4	3	Psychiatric diagnosis	16–40	ILA	Trial: mood disorders and other psychiatric illnesses requiring antenatal treatment	Multiple pregnancies, fetal malformations, chromosomal abnormalities, perinatal asphyxia, and dystocia	Positive correlation between amniotic fluid S100B and paroxetine concentrations in blood. Paroxetine-exposed pregnancies resulting in abnormal neonatal outcomes demonstrated the highest S100B levels
[59]	2016	trial: 53 control: 80	trial 26.33 ± 4.99 control 27.06 ± 6.06	3	SCID-I, BDI, BAI	trial: 32.85 ± 4.64 control: 33.06 ± 3.31	The AF was obtained by visually dividing the abdomen into four equal quadrants, measuring the deepest pocket (devoid of fetal parts or cord) and then adding them together.	NS	Maternal age 17 years or lower, existence of medical illnesses or pregnancy-related complications, existence of maternal infection, existence of any fetal malformation, using any psychotropic medication during pregnancy, smoking or alcohol consumption	Pregnant women diagnosed with oligohydramnios exhibited significantly higher scores for depressive and anxiety symptoms, alongside a higher prevalence of major depressive disorder and generalized anxiety disorder. In the majority of subjects, these clinical diagnoses preceded the detection of reduced amniotic fluid volume. These psychiatric conditions were more frequently observed in cases of severe oligohydramnios compared to moderate cases.
[56]	2015	trial: 75 control: 231	trial: 30 ± 5 control: 26 ± 4	3	Psychiatric diagnosis	At delivery–vaginal and cesarean section delivery trial: 38 ± 2 control: 40 ± 1	ILA	Presence of mood disorders or other psychiatric illnesses requiring antenatal treatment with SSRI; administration of paroxetine for at least 6 months	Multiple pregnancies, fetal malformations, chromosomal abnormalities, perinatal asphyxia, dystocia	Positive correlation between S100B level in maternal blood, amniotic fluid, both arterial and venous umbilical cord blood and SSRI administration. Highest concentrations were observed in neonates exhibiting neurological symptoms.
[58]	2011	trial: 24 control: 24	trial 30 ± 5 control 26 ± 4	3	Psychiatric diagnosis	At delivery–vaginal and caesarean section delivery trial: 39 ± 2 control:40 ± 1	EI	NS	Multiple pregnancies, fetal malformations, chromosomal abnormalities, perinatal asphyxia, dystocia during labor	Positive correlation between Activin A in both maternal and fetal biological fluids and SSRI administration.

Abbreviations: ILA—Immunoluminometry Assay; AF—Amniotic Fluid; BAI—Beck Anxiety Inventory; BDI—Beck Depression Inventory; EI—Enzyme Immunoassay; NS—Not Specified; SCID-I—Structured Clinical Interview for DSM-IV Axis I Disorders; SSRI—Selective Serotonin Reuptake Inhibitors.

**Table 3:** Impact of smoking on amniotic fluid composition.

Ref.	Year	Sample Size	Maternal Age [Years]	Pregnancy Trimesters	Smoking Status	Time of Sample Collection [Week]	Method of Analysis	Inclusion Criteria	Exclusion Criteria	Results
[61]	2017	81	34.4 ± 12	2	High-level nicotine exposure (>10 ng/mL) Low-level exposure (2–10 ng/mL), Minimal to no exposure (<2 ng/mL)	20.0 ± 3.7	HILIC-MS/MS	Confirmed normal fetal karyotype, paired maternal serum and amniotic fluid collected in the second trimester	Active smoking status, ambiguous exposure classification due to borderline cotinine levels.	Correlation between the nicotine exposure and depleted levels of acetylated polyamines, including diacetylspermine, acetylspermidine, and acetylspermine Increased concentrations of L-arginine and AMP, occurring alongside a marked reduction in proline, thymidine, and cytosine levels.
[62]	2012	222	35.3 ± 4.6	2	Declared smoking prior to AF collection; unable to determine blood/urine nicotine levels	15–18	ELISA (adiponectin), automated immunochemiluminescent assay (leptin, PAPP-A)	Singleton pregnancy, normal fetal anatomy, normal karyotype	Maternal hypertension, gestational diabetes, loss to follow-up in hospital, detection of a congenital abnormality	Negative correlation between maternal smoking status and adiponectin levels. Positive correlation between adiponectin levels and both insulin and PAPP-A.
[60]	2012	trial: 20 control: 67	trial 28.81 ± 6.9 control 33.24 ± 4.8	3	At least five cigarettes per day during pregnancy	At delivery trial 38.37 ± 1.81 control 38.38 ± 2.14	Total Antioxidant Capacity (TAC) Assay Kit, Malon dialdehyde (MDA) Adduct ELISA Kit.	NS	NS	MDA was higher in smokers than in non-smokers. TAC was lower in the amniotic fluid of smokers than in non-smokers.

Abbreviations: HILIC-MS/MS—Hydrophilic Interaction Chromatography with Tandem Mass Spectrometry; AMP—adenosine monophosphate; ELISA—Enzyme-Linked Immunosorbent Assay; PAPP-A—Pregnancy-Associated Plasma Protein A; MDA—Malon dialdehyde; TAC—Total Antioxidant Capacity.

**Table 4:** Impact of metabolic stressors on amniotic fluid composition.

Ref.	Year	Sample Size	Maternal Age [Years]	Pregnancy Trimesters	Maternal Factor	Time of Sample Collection [Week]	Method of Analysis	Inclusion Criteria	Exclusion Criteria	Results
[70]	2022	250	AF-BDNF not detectable: 33.5 ± 5.2 AF-BDNF detectable: 32.8 ± 5.4	3	Pre-pregnancy BMI	AF-BDNF not detectable: 32.2 ± 8.5 AF-BDNF detectable: 31.0 ± 8.6	BDNF Simplex ProcartaPlex Kit-immunoassay	Patients with singleton or twin pregnancies presenting to the ward for diagnostic amniocentesis, fetal therapy (laser coagulation of fetal anastomoses in twin-to-twin transfusion syndrome (TTTS), fetal tracheal occlusion with balloon catheter (FETO) or before C/S	Unwillingness to take part in the study, higher order multiple pregnancies, premature rupture of membranes, blood-stained AF samples, mothers with clinical signs of infection (fever >38 °C, leukocyte count > 15 G/L)	Positive correlation between maternal blood and amniotic fluid BDNF concentrations. No correlation between maternal and fetal blood BDNF levels. Negative correlation between maternal BMI before pregnancy and maternal BDNF.
[65]	2015	267	18–42	3	Diabetes	At delivery–cesarean section Control 25.3–41.9 (38.7) PE 24.9–41.4 (33.9) DM 32.7–42 (39) DPE 29.1–38.4 (35.9)	IA (IGF-15)	Singleton pregnancy	Pre-existing hypertension, rupture of membranes, clinical signs of infection	Amniotic fluid concentrations of growth differentiation factor 15 (GDF-15) were significantly elevated in pregnancies complicated by preeclampsia (PE) and diabetic preeclampsia (DPE) relative to healthy controls
[67]	2014	362	group 1 (IgM/IgA): 31.22 ± 4.61, group 2 (IgG): 29.59 ± 5.12, control: 28.60 ± 4.12	3	CMV	After piercing the fetal bladder during vaginal labor group 1 (IgM/IgA): 38.72 ± 4.61, group 2 (IgG): 38.51 ± 2.04, control: 38.60 ± 2.36	competitive ELISA	presence of anti-HCMV IgG/IgM	Evidence of inflammation, diabetic samples, samples contaminated with blood	Active cytomegalovirus infection was associated with reduced concentrations of 3-nitrotyrosine. This may suggest a compensatory upregulation of antioxidant defense mechanisms in this patient cohort
[68]	2014	trial 8, control 8	trial 31–46 (37.13), control 23–39 (33.88)	2	trial: BMI>30 at the time of amniocentesis control: BMI<25 at the time of amniocentesis	trial: 16–20 (17.86) sample: 16–21(18)	Extraction of cell-free fetal RNA from amniotic fluid and hybridization to whole-genome expression arrays	BMI fitting within stated range	Abnormal karyotype, RNA or cDNA of insufficient quality or quantity to hybridize to microarrays	205 significantly differentially regulated genes were identified in obese pregnant women, including a nine-fold upregulation of Apolipoprotein D. Downregulation of pathways associated with apoptotic cell death—specifically those involving the cerebral cortex and predicted activation of transcriptional regulators, including the estrogen receptor, FOS, and STAT3. This may suggest the pro-estrogenic and pro-inflammatory character of the intrauterine environment
[69]	2014	52	35.44 ± 3.58	2	Pre-pregnancy BMI	19.96 ± 1.95	Hexokinase method (glucose), Uricase method (uric acid), Molybdate reaction (phosphate), Ion-specific electrode (potassium, sodium)	Singleton pregnancy, absence of structural malformations and chromosomal abnormalities of the fetus, delivery of an appropriate for gestational age infant (birth weight between the 10th and 90th centile), absence of obstetrical or medical complications, such as preeclampsia or diabetes mellitus	Not meeting all inclusion criteria	Positive correlation between maternal pre-pregnancy BMI and amniotic fluid uric acid concentrations. Marginal associations between maternal BMI and both amniotic fluid glucose and sodium levels.
[66]	1987	55	NS	NS	Diabetes	19–42	double antibody RIA	NS	NS	Maternal comorbidities, including diabetes mellitus and hypertension, did not result in an elevation of NSE concentrations within the amniotic fluid

Values in parentheses represent mean maternal or gestational age. Abbreviations: AF—Amniotic Fluid; BDNF—Brain-Derived Neurotrophic Factor; BMI—Body Mass Index; cDNA—complementary deoxyribonucleic Acid; CMV—cytomegalovirus; DPE—diabetic preeclampsia; ELISA—Enzyme-Linked Immunosorbent Assay; FETO—fetal tracheal occlusion; FOS—FBJ murine osteosarcoma viral oncogene homolog; GDF-15—Growth-Differentiation Factor 15; HCMV—Human Cytomegalovirus; IA—Immunoassay; IGF—Insulin-like Growth Factor; NSE—neuron-specific enolase; NS—Not Specified; PE—preeclampsia; RIA—Radioimmunoassay; RNA—ribonucleic acid; STAT3—Signal Transducer and Activator of Transcription 3; TTTS—twin-to-twin transfusion syndrome.

## Data Availability

No new data were created or analyzed in this study. Data sharing is not applicable to this article.

## Supplementary Materials

The following supporting information can be downloaded at https://www.mdpi.com/article/10.3390/ijms27188434/s1.

## Abbreviations

The following abbreviations are used in this manuscript:

11β-HSD2	11β Hydroxysteroid Dehydrogenase 2
ACTH	Adrenocorticotropic Hormone
ADHD	Attention Deficit Hyperactivity Disorder
AF	Amniotic Fluid
AGA	Appropriate for Gestational Age
AMP	adenosine monophosphate
ANS	Autonomic Nervous System
anti-HCMG	Anti-Human Cytomegalovirus Antibodies
BAI	Beck Anxiety Inventory
BDI	Beck Depression Inventory
BDNF	Brain-Derived Neurotrophic Factor
BMI	Body Mass Index
CAPSC	Clinician-Administered PTSD Scale
cDNA	complementary Deoxyribonucleic Acid
CMV	cytomegalovirus
CRH	Corticotropin-Releasing Hormone
CSS	Chronic Stress Scale
CTQ	Childhood Trauma Questionnaire
DES	Dissociative Experiences Scale
DPE	Diabetic Preeclampsia
EI	Enzyme Immunoassay
ELISA	Enzyme-Linked Immunosorbent Assay
EPDS	Edinburgh Postnatal Depression Scale
FASD	Fetal Alcohol Spectrum Disorders
FETO	Fetal Tracheal Occlusion
FOS	FBJ murine osteosarcoma viral oncogene homolog
F-Sozu	Questionnaires on Social Support
GDF-15	Growth-Differentiation Factor 15
HILIC-MS/MS	Hydrophilic Interaction Chromatography with Tandem Mass Spectrometry
HIV	Human Immunodeficiency Virus
HPLC-UV	High-Performance Liquid Chromatography with UV-Vis Detection
IA	Immunoassay
IGF	Insulin-like Growth Factor
IL-10	interleukin-10
IL-6	interleukin-6
ILA	Immunoluminometry Assay
IRMAG	Interview for Maternal Representations During Pregnancy
LCMS	Liquid Chromatography–Mass Spectrometry
LDL	Low-Density Lipoprotein
MDA	Malondialdehyde
MDMQ	Multidimensional Mood State Questionnaire
MINI	Mini-International Neuropsychiatric Interview
MPO	Myeloperoxidase
NIH	National Institutes of Health
NSE	Neuron-Specific Enolase
NS	Not Specified
PAPP-A	Pregnancy-Associated Plasma Protein A
PA	Prenatal Attachment to the Offspring
PDQ	Personality Diagnostic Questionnaire
PE	Preeclampsia
PPD	Postpartum Depression
PPQ	Perinatal PTSD Questionnaire
PSS	Perceived Stress Scale
RIA	Radioimmunoassay
RNA	ribonucleic acid
SCID-I	Structured Clinical Interview for DSM-IV Axis I Disorders
SGA	Small for Gestational Age
SLEQ	Sleep Experience Questionnaire
SSRI	Selective Serotonin Reuptake Inhibitors
STAI	State-Trait Anxiety Inventory
STAT3	Signal Transducer and Activator of Transcription 3
T3	triiodothyronine
T4	tetraiodothyronine
TAC	Total Antioxidant Capacity
TICS	Trier Inventory for Chronic Stress
TNF-α	Tumor Necrosis Factor α
TORCH	Toxoplasmosis, Others, Rubella, Cytomegalovirus, Herpes
TSH	Thyroid-Stimulating Hormone
TTTS	Twin-to-Twin Transfusion Syndrome
VAS	Visual Analog Scale
