# Supplementary material for: Stressed, Stained, and Stored: The Impact of Maternal Distress and Smoking on Amniotic Biomarkers—A Scoping Review
Source: Int J Mol Sci. 2026 Sep 21;27(18):8434. doi: 10.3390/ijms27188434 (PMC13607953; doi:10.3390/ijms27188434)
Supplement: Supplementary file 1 [file ijms-27-08434-s001.zip › ijms-4504701-supplementary.pdf]

Supplementary material for "Stressed, Stained, and Stored: The Impact of Maternal Distress and Smoking on Amniotic Biomarkers – scoping review"

Content:

1. Search strategy………2
2. Quality assessment – mental state……………………………………………………………………………………3
3. Quality assessment – psychiatric diagnosis and treatment…………………………………………………..20
4. Quality assessment – smoking……………………………………………………………………………………………25
5. Quality assessment – other………………………………..………………………………………………………………28

1. Search strategy

Systematic search

SEARCH	BASE	FILTERS	N=
('amniotic fluid' OR 'amniotic fluid composition' OR 'amniotic biochemistry') AND ('psychological stress' OR 'maternal stress' OR 'anxiety' OR 'depression' OR 'psychological factors')	PubMed	HUMAN	528
('amniotic fluid'/exp OR 'amniotic fluid composition' OR 'amniotic biochemistry') AND ('psychological stress'/exp OR 'maternal stress' OR 'anxiety'/exp OR 'depression'/exp OR 'psychological factors')	EMBASE	NO	263
('amniotic fluid' OR 'amniotic fluid composition' OR 'amniotic biochemistry') AND ('psychological stress' OR 'maternal stress' OR 'anxiety' OR 'depression' OR 'psychological factors')	WebOfScience	NO	574

Additional search:

- Above mentioned – searching for new position
- Using above mentioned key words in Scholar
- Screening reviews obtained in systematic search process
- Screening authors with more than one study already included

2. Quality assessment – mental state

Criteria: „Prenatal Cortisol Exposure Predicts Infant Cortisol Response to Acute Stress"	Yes	No	Other (CD, NR, NA)*
1. Was the research question or objective in this paper clearly stated?	x		
2. Was the study population clearly specified and defined?	X		
3. Was the participation rate of eligible persons at least 50%?		x	
4. Were all the subjects selected or recruited from the same or similar populations (including the same time period)? Were inclusion and exclusion criteria for being in the study prespecified and applied uniformly to all participants?	X		
5. Was a sample size justification, power description, or variance and effect estimates provided?		x	
6. For the analyses in this paper, were the exposure(s) of interest measured prior to the outcome(s) being measured?	X		
7. Was the timeframe sufficient so that one could reasonably expect to see an association between exposure and outcome if it existed?	X		
8. For exposures that can vary in amount or level, did the study examine different levels of the exposure as related to the outcome (e.g., categories of exposure, or exposure measured as continuous variable)?	X		
9. Were the exposure measures (independent variables) clearly defined, valid, reliable, and implemented consistently across all study participants?	X		
10. Was the exposure(s) assessed more than once over time?		x	
11. Were the outcome measures (dependent variables) clearly defined, valid, reliable, and implemented consistently across all study participants?	X		
12. Were the outcome assessors blinded to the exposure status of participants?			NR
13. Was loss to follow-up after baseline 20% or less?		X	
14. Were key potential confounding variables measured and adjusted statistically for their impact on the relationship between exposure(s) and outcome(s)?	X		

Criteria: „The Association Between the Acute Psychobiological Stress Response in Second Trimester Pregnant Women, Amniotic Fluid Glucocorticoids, and Neonatal Birth Outcome”	Yes	No	Other (CD, NR, NA)*
1. Was the research question or objective in this paper clearly stated?	X		
2. Was the study population clearly specified and defined?	X		
3. Was the participation rate of eligible persons at least 50%?		x	
4. Were all the subjects selected or recruited from the same or similar populations (including the same time period)? Were inclusion and exclusion criteria for being in the study prespecified and applied uniformly to all participants?	X		
5. Was a sample size justification, power description, or variance and effect estimates provided?		x	
6. For the analyses in this paper, were the exposure(s) of interest measured prior to the outcome(s) being measured?	X		
7. Was the timeframe sufficient so that one could reasonably expect to see an association between exposure and outcome if it existed?	X		
8. For exposures that can vary in amount or level, did the study examine different levels of the exposure as related to the outcome (e.g., categories of exposure, or exposure measured as continuous variable)?	X		
9. Were the exposure measures (independent variables) clearly defined, valid, reliable, and implemented consistently across all study participants?	X		
10. Was the exposure(s) assessed more than once over time?	X		
11. Were the outcome measures (dependent variables) clearly defined, valid, reliable, and implemented consistently across all study participants?	X		
12. Were the outcome assessors blinded to the exposure status of participants?			NR
13. Was loss to follow-up after baseline 20% or less?	X		
14. Were key potential confounding variables measured and adjusted statistically for their impact on the relationship between exposure(s) and outcome(s)?	x		

**Criteria: „Maternal anxiety and fetal behavior at 15 weeks' gestation"**	**Yes**	**No**	**Other (CD, NR, NA)***
1. Was the research question or objective in this paper clearly stated?	X		
2. Was the study population clearly specified and defined?	X		
3. Was the participation rate of eligible persons at least 50%?			CD
4. Were all the subjects selected or recruited from the same or similar populations (including the same time period)? Were inclusion and exclusion criteria for being in the study prespecified and applied uniformly to all participants?	X		
5. Was a sample size justification, power description, or variance and effect estimates provided?		x	
6. For the analyses in this paper, were the exposure(s) of interest measured prior to the outcome(s) being measured?	X		
7. Was the timeframe sufficient so that one could reasonably expect to see an association between exposure and outcome if it existed?	X		
8. For exposures that can vary in amount or level, did the study examine different levels of the exposure as related to the outcome (e.g., categories of exposure, or exposure measured as continuous variable)?	X		
9. Were the exposure measures (independent variables) clearly defined, valid, reliable, and implemented consistently across all study participants?	X		
10. Was the exposure(s) assessed more than once over time?		x	
11. Were the outcome measures (dependent variables) clearly defined, valid, reliable, and implemented consistently across all study participants?	X		
12. Were the outcome assessors blinded to the exposure status of participants?	X		
13. Was loss to follow-up after baseline 20% or less?	X		
14. Were key potential confounding variables measured and adjusted statistically for their impact on the relationship between exposure(s) and outcome(s)?		x	

**Criteria „Maternal stress and fetoplacental cortisol regulation in small-for-gestational-age newborns"**	**Yes**	**No**	**Other (CD, NR, NA)***
1. Was the research question or objective in this paper clearly stated?	X		
2. Was the study population clearly specified and defined?	X		
3. Was the participation rate of eligible persons at least 50%?	X		
4. Were all the subjects selected or recruited from the same or similar populations (including the same time period)? Were inclusion and exclusion criteria for being in the study prespecified and applied uniformly to all participants?	X		
5. Was a sample size justification, power description, or variance and effect estimates provided?		x	
6. For the analyses in this paper, were the exposure(s) of interest measured prior to the outcome(s) being measured?	X		
7. Was the timeframe sufficient so that one could reasonably expect to see an association between exposure and outcome if it existed?	X		
8. For exposures that can vary in amount or level, did the study examine different levels of the exposure as related to the outcome (e.g., categories of exposure, or exposure measured as continuous variable)?	X		
9. Were the exposure measures (independent variables) clearly defined, valid, reliable, and implemented consistently across all study participants?	X		
10. Was the exposure(s) assessed more than once over time?		x	
11. Were the outcome measures (dependent variables) clearly defined, valid, reliable, and implemented consistently across all study participants?	X		
12. Were the outcome assessors blinded to the exposure status of participants?			NR
13. Was loss to follow-up after baseline 20% or less?	X		
14. Were key potential confounding variables measured and adjusted statistically for their impact on the relationship between exposure(s) and outcome(s)?	x		

Criteria „Maternal Prenatal Cortisol and Infant Cognitive Development: Moderation by Infant–Mother Attachment”	Yes	No	Other (CD, NR, NA)*
1. Was the research question or objective in this paper clearly stated?	X		
2. Was the study population clearly specified and defined?	X		
3. Was the participation rate of eligible persons at least 50%?			CD
4. Were all the subjects selected or recruited from the same or similar populations (including the same time period)? Were inclusion and exclusion criteria for being in the study prespecified and applied uniformly to all participants?	X		
5. Was a sample size justification, power description, or variance and effect estimates provided?		x	
6. For the analyses in this paper, were the exposure(s) of interest measured prior to the outcome(s) being measured?	X		
7. Was the timeframe sufficient so that one could reasonably expect to see an association between exposure and outcome if it existed?	X		
8. For exposures that can vary in amount or level, did the study examine different levels of the exposure as related to the outcome (e.g., categories of exposure, or exposure measured as continuous variable)?	X		
9. Were the exposure measures (independent variables) clearly defined, valid, reliable, and implemented consistently across all study participants?	X		
10. Was the exposure(s) assessed more than once over time?		x	
11. Were the outcome measures (dependent variables) clearly defined, valid, reliable, and implemented consistently across all study participants?	X		
12. Were the outcome assessors blinded to the exposure status of participants?	X		
13. Was loss to follow-up after baseline 20% or less?	X		
14. Were key potential confounding variables measured and adjusted statistically for their impact on the relationship between exposure(s) and outcome(s)?	X		

Criteria „Association between maternal and amniotic fluid cortisol is moderated by maternal anxiety”	Yes	No	Other (CD, NR, NA)*
1. Was the research question or objective in this paper clearly stated?	x		
2. Was the study population clearly specified and defined?	x		
3. Was the participation rate of eligible persons at least 50%?	x		
4. Were all the subjects selected or recruited from the same or similar populations (including the same time period)? Were inclusion and exclusion criteria for being in the study prespecified and applied uniformly to all participants?	x		
5. Was a sample size justification, power description, or variance and effect estimates provided?		x	
6. For the analyses in this paper, were the exposure(s) of interest measured prior to the outcome(s) being measured?		x	
7. Was the timeframe sufficient so that one could reasonably expect to see an association between exposure and outcome if it existed?		x	
8. For exposures that can vary in amount or level, did the study examine different levels of the exposure as related to the outcome (e.g., categories of exposure, or exposure measured as continuous variable)?	x		
9. Were the exposure measures (independent variables) clearly defined, valid, reliable, and implemented consistently across all study participants?	x		
10. Was the exposure(s) assessed more than once over time?		x	
11. Were the outcome measures (dependent variables) clearly defined, valid, reliable, and implemented consistently across all study participants?	x		
12. Were the outcome assessors blinded to the exposure status of participants?			NR
13. Was loss to follow-up after baseline 20% or less?	x		
14. Were key potential confounding variables measured and adjusted statistically for their impact on the relationship between exposure(s) and outcome(s)?	x		

Criteria „Influence of prenatal maternal stress, maternal plasma cortisol and cortisol in the amniotic fluid on birth outcomes and child temperament at 3 months”	Yes	No	Other (CD, NR, NA)*
1. Was the research question or objective in this paper clearly stated?	x		
2. Was the study population clearly specified and defined?	x		
3. Was the participation rate of eligible persons at least 50%?	x		
4. Were all the subjects selected or recruited from the same or similar populations (including the same time period)? Were inclusion and exclusion criteria for being in the study prespecified and applied uniformly to all participants?	x		
5. Was a sample size justification, power description, or variance and effect estimates provided?		x	
6. For the analyses in this paper, were the exposure(s) of interest measured prior to the outcome(s) being measured?	x		
7. Was the timeframe sufficient so that one could reasonably expect to see an association between exposure and outcome if it existed?	x		
8. For exposures that can vary in amount or level, did the study examine different levels of the exposure as related to the outcome (e.g., categories of exposure, or exposure measured as continuous variable)?	x		
9. Were the exposure measures (independent variables) clearly defined, valid, reliable, and implemented consistently across all study participants?	x		
10. Was the exposure(s) assessed more than once over time?		x	
11. Were the outcome measures (dependent variables) clearly defined, valid, reliable, and implemented consistently across all study participants?	x		
12. Were the outcome assessors blinded to the exposure status of participants?			NR
13. Was loss to follow-up after baseline 20% or less?	x		
14. Were key potential confounding variables measured and adjusted statistically for their impact on the relationship between exposure(s) and outcome(s)?	x		

Criteria “Childhood trauma and insulin-like growth factors in amniotic fluid – An exploratory analysis of 79 women”	Yes	No	Other (CD, NR, NA)*
1. Was the research question or objective in this paper clearly stated?	x		
2. Was the study population clearly specified and defined?	x		
3. Was the participation rate of eligible persons at least 50%?	x		
4. Were all the subjects selected or recruited from the same or similar populations (including the same time period)? Were inclusion and exclusion criteria for being in the study prespecified and applied uniformly to all participants?	x		
5. Was a sample size justification, power description, or variance and effect estimates provided?	x		
6. For the analyses in this paper, were the exposure(s) of interest measured prior to the outcome(s) being measured?	x		
7. Was the timeframe sufficient so that one could reasonably expect to see an association between exposure and outcome if it existed?	x		
8. For exposures that can vary in amount or level, did the study examine different levels of the exposure as related to the outcome (e.g., categories of exposure, or exposure measured as continuous variable)?	x		
9. Were the exposure measures (independent variables) clearly defined, valid, reliable, and implemented consistently across all study participants?	x		
10. Was the exposure(s) assessed more than once over time?		x	
11. Were the outcome measures (dependent variables) clearly defined, valid, reliable, and implemented consistently across all study participants?	x		
12. Were the outcome assessors blinded to the exposure status of participants?			NR
13. Was loss to follow-up after baseline 20% or less?	x		
14. Were key potential confounding variables measured and adjusted statistically for their impact on the relationship between exposure(s) and outcome(s)?	x		

Criteria "In utero cortisol and testosterone exposure and fear reactivity in infancy"	Yes	No	Other (CD, NR, NA)*
1. Was the research question or objective in this paper clearly stated?	x		
2. Was the study population clearly specified and defined?	x		
3. Was the participation rate of eligible persons at least 50%?	x		
4. Were all the subjects selected or recruited from the same or similar populations (including the same time period)? Were inclusion and exclusion criteria for being in the study prespecified and applied uniformly to all participants?	x		
5. Was a sample size justification, power description, or variance and effect estimates provided?		x	
6. For the analyses in this paper, were the exposure(s) of interest measured prior to the outcome(s) being measured?	x		
7. Was the timeframe sufficient so that one could reasonably expect to see an association between exposure and outcome if it existed?	x		
8. For exposures that can vary in amount or level, did the study examine different levels of the exposure as related to the outcome (e.g., categories of exposure, or exposure measured as continuous variable)?	x		
9. Were the exposure measures (independent variables) clearly defined, valid, reliable, and implemented consistently across all study participants?	x		
10. Was the exposure(s) assessed more than once over time?		x	
11. Were the outcome measures (dependent variables) clearly defined, valid, reliable, and implemented consistently across all study participants?	x		
12. Were the outcome assessors blinded to the exposure status of participants?	x		
13. Was loss to follow-up after baseline 20% or less?	x		
14. Were key potential confounding variables measured and adjusted statistically for their impact on the relationship between exposure(s) and outcome(s)?	x		

Criteria “Stress in pregnancy – Implications for fetal BDNF in amniotic fluid at birth”	Yes	No	Other (CD, NR, NA)*
1. Was the research question or objective in this paper clearly stated?	x		
2. Was the study population clearly specified and defined?	x		
3. Was the participation rate of eligible persons at least 50%?	x		
4. Were all the subjects selected or recruited from the same or similar populations (including the same time period)? Were inclusion and exclusion criteria for being in the study prespecified and applied uniformly to all participants?	x		
5. Was a sample size justification, power description, or variance and effect estimates provided?	x		
6. For the analyses in this paper, were the exposure(s) of interest measured prior to the outcome(s) being measured?	x		
7. Was the timeframe sufficient so that one could reasonably expect to see an association between exposure and outcome if it existed?	x		
8. For exposures that can vary in amount or level, did the study examine different levels of the exposure as related to the outcome (e.g., categories of exposure, or exposure measured as continuous variable)?	x		
9. Were the exposure measures (independent variables) clearly defined, valid, reliable, and implemented consistently across all study participants?	x		
10. Was the exposure(s) assessed more than once over time?		x	
11. Were the outcome measures (dependent variables) clearly defined, valid, reliable, and implemented consistently across all study participants?	x		
12. Were the outcome assessors blinded to the exposure status of participants?			NR
13. Was loss to follow-up after baseline 20% or less?	x		
14. Were key potential confounding variables measured and adjusted statistically for their impact on the relationship between exposure(s) and outcome(s)?	x		

Criteria “Cortisol, cortisone, and BDNF in amniotic fluid in the second trimester of pregnancy: Effect of early life and current maternal stress and socioeconomic status”	Yes	No	Other (CD, NR, NA)*
1. Was the research question or objective in this paper clearly stated?	x		
2. Was the study population clearly specified and defined?	x		
3. Was the participation rate of eligible persons at least 50%?	x		
4. Were all the subjects selected or recruited from the same or similar populations (including the same time period)? Were inclusion and exclusion criteria for being in the study prespecified and applied uniformly to all participants?	x		
5. Was a sample size justification, power description, or variance and effect estimates provided?		x	
6. For the analyses in this paper, were the exposure(s) of interest measured prior to the outcome(s) being measured?		x	
7. Was the timeframe sufficient so that one could reasonably expect to see an association between exposure and outcome if it existed?		x	
8. For exposures that can vary in amount or level, did the study examine different levels of the exposure as related to the outcome (e.g., categories of exposure, or exposure measured as continuous variable)?	x		
9. Were the exposure measures (independent variables) clearly defined, valid, reliable, and implemented consistently across all study participants?	x		
10. Was the exposure(s) assessed more than once over time?		x	
11. Were the outcome measures (dependent variables) clearly defined, valid, reliable, and implemented consistently across all study participants?	x		
12. Were the outcome assessors blinded to the exposure status of participants?			NR
13. Was loss to follow-up after baseline 20% or less?			NA
14. Were key potential confounding variables measured and adjusted statistically for their impact on the relationship between exposure(s) and outcome(s)?	x		

Criteria “Second-Trimester Amniotic Fluid Corticotropin-Releasing Hormone and Urocortin in Relation to Maternal Stress and Fetal Growth in Human Pregnancy”	Yes	No	Other (CD, NR, NA)*
1. Was the research question or objective in this paper clearly stated?	x		
2. Was the study population clearly specified and defined?	x		
3. Was the participation rate of eligible persons at least 50%?	x		
4. Were all the subjects selected or recruited from the same or similar populations (including the same time period)? Were inclusion and exclusion criteria for being in the study prespecified and applied uniformly to all participants?	x		
5. Was a sample size justification, power description, or variance and effect estimates provided?		x	
6. For the analyses in this paper, were the exposure(s) of interest measured prior to the outcome(s) being measured?	x		
7. Was the timeframe sufficient so that one could reasonably expect to see an association between exposure and outcome if it existed?	x		
8. For exposures that can vary in amount or level, did the study examine different levels of the exposure as related to the outcome (e.g., categories of exposure, or exposure measured as continuous variable)?	x		
9. Were the exposure measures (independent variables) clearly defined, valid, reliable, and implemented consistently across all study participants?	x		
10. Was the exposure(s) assessed more than once over time?	x		
11. Were the outcome measures (dependent variables) clearly defined, valid, reliable, and implemented consistently across all study participants?	x		
12. Were the outcome assessors blinded to the exposure status of participants?			NR
13. Was loss to follow-up after baseline 20% or less?	x		
14. Were key potential confounding variables measured and adjusted statistically for their impact on the relationship between exposure(s) and outcome(s)?	x		

Criteria "The Acute Autonomic Stress Response and Amniotic Fluid Glucocorticoids in Second-Trimester Pregnant Women"	Yes	No	Other (CD, NR, NA)*
1. Was the research question or objective in this paper clearly stated?	x		
2. Was the study population clearly specified and defined?	x		
3. Was the participation rate of eligible persons at least 50%?	x		
4. Were all the subjects selected or recruited from the same or similar populations (including the same time period)? Were inclusion and exclusion criteria for being in the study prespecified and applied uniformly to all participants?	x		
5. Was a sample size justification, power description, or variance and effect estimates provided?		x	
6. For the analyses in this paper, were the exposure(s) of interest measured prior to the outcome(s) being measured?	x		
7. Was the timeframe sufficient so that one could reasonably expect to see an association between exposure and outcome if it existed?	x		
8. For exposures that can vary in amount or level, did the study examine different levels of the exposure as related to the outcome (e.g., categories of exposure, or exposure measured as continuous variable)?	x		
9. Were the exposure measures (independent variables) clearly defined, valid, reliable, and implemented consistently across all study participants?	x		
10. Was the exposure(s) assessed more than once over time?	x		
11. Were the outcome measures (dependent variables) clearly defined, valid, reliable, and implemented consistently across all study participants?	x		
12. Were the outcome assessors blinded to the exposure status of participants?			NR
13. Was loss to follow-up after baseline 20% or less?			NA
14. Were key potential confounding variables measured and adjusted statistically for their impact on the relationship between exposure(s) and outcome(s)?	x		

Criteria “Ontogeny of foetal exposure to maternal cortisol using midtrimester amniotic fluid as a biomarker”	Yes	No	Other (CD, NR, NA)*
1. Was the research question or objective in this paper clearly stated?	x		
2. Was the study population clearly specified and defined?	x		
3. Was the participation rate of eligible persons at least 50%?	x		
4. Were all the subjects selected or recruited from the same or similar populations (including the same time period)? Were inclusion and exclusion criteria for being in the study prespecified and applied uniformly to all participants?	x		
5. Was a sample size justification, power description, or variance and effect estimates provided?		x	
6. For the analyses in this paper, were the exposure(s) of interest measured prior to the outcome(s) being measured?	x		
7. Was the timeframe sufficient so that one could reasonably expect to see an association between exposure and outcome if it existed?	x		
8. For exposures that can vary in amount or level, did the study examine different levels of the exposure as related to the outcome (e.g., categories of exposure, or exposure measured as continuous variable)?	x		
9. Were the exposure measures (independent variables) clearly defined, valid, reliable, and implemented consistently across all study participants?	x		
10. Was the exposure(s) assessed more than once over time?		x	
11. Were the outcome measures (dependent variables) clearly defined, valid, reliable, and implemented consistently across all study participants?	x		
12. Were the outcome assessors blinded to the exposure status of participants?			NR
13. Was loss to follow-up after baseline 20% or less?			NA
14. Were key potential confounding variables measured and adjusted statistically for their impact on the relationship between exposure(s) and outcome(s)?	x		

Criteria "Amniotic fluid testosterone: relationship with cortisol and gestational age"	Yes	No	Other (CD, NR, NA)*
1. Was the research question or objective in this paper clearly stated?	x		
2. Was the study population clearly specified and defined?	x		
3. Was the participation rate of eligible persons at least 50%?	x		
4. Were all the subjects selected or recruited from the same or similar populations (including the same time period)? Were inclusion and exclusion criteria for being in the study prespecified and applied uniformly to all participants?	x		
5. Was a sample size justification, power description, or variance and effect estimates provided?		x	
6. For the analyses in this paper, were the exposure(s) of interest measured prior to the outcome(s) being measured?	x		
7. Was the timeframe sufficient so that one could reasonably expect to see an association between exposure and outcome if it existed?	x		
8. For exposures that can vary in amount or level, did the study examine different levels of the exposure as related to the outcome (e.g., categories of exposure, or exposure measured as continuous variable)?	x		
9. Were the exposure measures (independent variables) clearly defined, valid, reliable, and implemented consistently across all study participants?	x		
10. Was the exposure(s) assessed more than once over time?		x	
11. Were the outcome measures (dependent variables) clearly defined, valid, reliable, and implemented consistently across all study participants?	x		
12. Were the outcome assessors blinded to the exposure status of participants?			NR
13. Was loss to follow-up after baseline 20% or less?			NA
14. Were key potential confounding variables measured and adjusted statistically for their impact on the relationship between exposure(s) and outcome(s)?	x		

Criteria “Maternal Antenatal Anxiety and Amniotic Fluid Cortisol and Testosterone: Possible Implications for Foetal Programming”	Yes	No	Other (CD, NR, NA)*
1. Was the research question or objective in this paper clearly stated?	x		
2. Was the study population clearly specified and defined?	x		
3. Was the participation rate of eligible persons at least 50%?			NR
4. Were all the subjects selected or recruited from the same or similar populations (including the same time period)? Were inclusion and exclusion criteria for being in the study prespecified and applied uniformly to all participants?	x		
5. Was a sample size justification, power description, or variance and effect estimates provided?		x	
6. For the analyses in this paper, were the exposure(s) of interest measured prior to the outcome(s) being measured?	x		
7. Was the timeframe sufficient so that one could reasonably expect to see an association between exposure and outcome if it existed?	x		
8. For exposures that can vary in amount or level, did the study examine different levels of the exposure as related to the outcome (e.g., categories of exposure, or exposure measured as continuous variable)?	x		
9. Were the exposure measures (independent variables) clearly defined, valid, reliable, and implemented consistently across all study participants?	x		
10. Was the exposure(s) assessed more than once over time?		x	
11. Were the outcome measures (dependent variables) clearly defined, valid, reliable, and implemented consistently across all study participants?	x		
12. Were the outcome assessors blinded to the exposure status of participants?			NR
13. Was loss to follow-up after baseline 20% or less?			NA
14. Were key potential confounding variables measured and adjusted statistically for their impact on the relationship between exposure(s) and outcome(s)?	x		

Criteria “Maternal prenatal depression and anxiety and steroid hormones in amniotic fluid”	Yes	No	Other (CD, NR, NA)*
1. Was the research question or objective in this paper clearly stated?	x		
2. Was the study population clearly specified and defined?	x		
3. Was the participation rate of eligible persons at least 50%?			NR
4. Were all the subjects selected or recruited from the same or similar populations (including the same time period)? Were inclusion and exclusion criteria for being in the study prespecified and applied uniformly to all participants?	x		
5. Was a sample size justification, power description, or variance and effect estimates provided?		x	
6. For the analyses in this paper, were the exposure(s) of interest measured prior to the outcome(s) being measured?	x		
7. Was the timeframe sufficient so that one could reasonably expect to see an association between exposure and outcome if it existed?	x		
8. For exposures that can vary in amount or level, did the study examine different levels of the exposure as related to the outcome (e.g., categories of exposure, or exposure measured as continuous variable)?	x		
9. Were the exposure measures (independent variables) clearly defined, valid, reliable, and implemented consistently across all study participants?	x		
10. Was the exposure(s) assessed more than once over time?	x		
11. Were the outcome measures (dependent variables) clearly defined, valid, reliable, and implemented consistently across all study participants?	x		
12. Were the outcome assessors blinded to the exposure status of participants?			NR
13. Was loss to follow-up after baseline 20% or less?			NA
14. Were key potential confounding variables measured and adjusted statistically for their impact on the relationship between exposure(s) and outcome(s)?	x		

3. Quality assessment – psychiatric treatment

**Criteria Antenatal Maternal Antidepressants Drugs Treatment Affects S100B Levels in Maternal-Fetal Biological Fluids in a Dose Dependent Manner**	**Yes**	**No**	**Other (CD, NR, NA)***
1. Was the research question or objective in this paper clearly stated?	x		
2. Was the study population clearly specified and defined?	x		
3. Was the participation rate of eligible persons at least 50%?			CD
4. Were all the subjects selected or recruited from the same or similar populations (including the same time period)? Were inclusion and exclusion criteria for being in the study prespecified and applied uniformly to all participants?	x		
5. Was a sample size justification, power description, or variance and effect estimates provided?		x	
6. For the analyses in this paper, were the exposure(s) of interest measured prior to the outcome(s) being measured?		x	
7. Was the timeframe sufficient so that one could reasonably expect to see an association between exposure and outcome if it existed?	x		
8. For exposures that can vary in amount or level, did the study examine different levels of the exposure as related to the outcome (e.g., categories of exposure, or exposure measured as continuous variable)?	x		
9. Were the exposure measures (independent variables) clearly defined, valid, reliable, and implemented consistently across all study participants?	x		
10. Was the exposure(s) assessed more than once over time?	x		
11. Were the outcome measures (dependent variables) clearly defined, valid, reliable, and implemented consistently across all study participants?	x		
12. Were the outcome assessors blinded to the exposure status of participants?			NR
13. Was loss to follow-up after baseline 20% or less?	x		
14. Were key potential confounding variables measured and adjusted statistically for their impact on the relationship between exposure(s) and outcome(s)?	x		

Criteria Are Major Depression and Generalized Anxiety Disorder Associated With Oligohydramnios in Pregnant Women? A Case–Control Study	Yes	No	Other (CD, NR, NA)*
1. Was the research question or objective in this paper clearly stated and appropriate?	x		
2. Was the study population clearly specified and defined?	x		
3. Did the authors include a sample size justification?		x	
4. Were controls selected or recruited from the same or similar population that gave rise to the cases (including the same timeframe)?	x		
5. Were the definitions, inclusion and exclusion criteria, algorithms or processes used to identify or select cases and controls valid, reliable, and implemented consistently across all study participants?	x		
6. Were the cases clearly defined and differentiated from controls?	x		
7. If less than 100 percent of eligible cases and/or controls were selected for the study, were the cases and/or controls randomly selected from those eligible?			CD
8. Was there use of concurrent controls?	x		
9. Were the investigators able to confirm that the exposure/risk occurred prior to the development of the condition or event that defined a participant as a case?	x		
10. Were the measures of exposure/risk clearly defined, valid, reliable, and implemented consistently (including the same time period) across all study participants?	x		
11. Were the assessors of exposure/risk blinded to the case or control status of participants?	x		
12. Were key potential confounding variables measured and adjusted statistically in the analyses? If matching was used, did the investigators account for matching during study analysis?		x	

Criteria Antenatal Maternal Antidepressants Drugs Affect S100B Concentrations in Fetal-Maternal Biological Fluids	Yes	No	Other (CD, NR, NA)*
1. Was the research question or objective in this paper clearly stated?	x		
2. Was the study population clearly specified and defined?	x		
3. Was the participation rate of eligible persons at least 50%?			CD
4. Were all the subjects selected or recruited from the same or similar populations (including the same time period)? Were inclusion and exclusion criteria for being in the study prespecified and applied uniformly to all participants?	x		
5. Was a sample size justification, power description, or variance and effect estimates provided?		x	
6. For the analyses in this paper, were the exposure(s) of interest measured prior to the outcome(s) being measured?	x		
7. Was the timeframe sufficient so that one could reasonably expect to see an association between exposure and outcome if it existed?		x	
8. For exposures that can vary in amount or level, did the study examine different levels of the exposure as related to the outcome (e.g., categories of exposure, or exposure measured as continuous variable)?			NA
9. Were the exposure measures (independent variables) clearly defined, valid, reliable, and implemented consistently across all study participants?	x		
10. Was the exposure(s) assessed more than once over time?	x		
11. Were the outcome measures (dependent variables) clearly defined, valid, reliable, and implemented consistently across all study participants?	x		
12. Were the outcome assessors blinded to the exposure status of participants?			NR
13. Was loss to follow-up after baseline 20% or less?	x		
14. Were key potential confounding variables measured and adjusted statistically for their impact on the relationship between exposure(s) and outcome(s)?		x	

Criteria Antenatal maternal antidepressants drugs affect Activin A concentrations in maternal blood, in amniotic fluid and in fetal cord blood	Yes	No	Other (CD, NR, NA)*
1. Was the research question or objective in this paper clearly stated and appropriate?	x		
2. Was the study population clearly specified and defined?	x		
3. Did the authors include a sample size justification?		x	
4. Were controls selected or recruited from the same or similar population that gave rise to the cases (including the same timeframe)?	x		
5. Were the definitions, inclusion and exclusion criteria, algorithms or processes used to identify or select cases and controls valid, reliable, and implemented consistently across all study participants?	x		
6. Were the cases clearly defined and differentiated from controls?	x		
7. If less than 100 percent of eligible cases and/or controls were selected for the study, were the cases and/or controls randomly selected from those eligible?			CD
8. Was there use of concurrent controls?	x		
9. Were the investigators able to confirm that the exposure/risk occurred prior to the development of the condition or event that defined a participant as a case?	x		
10. Were the measures of exposure/risk clearly defined, valid, reliable, and implemented consistently (including the same time period) across all study participants?	x		
11. Were the assessors of exposure/risk blinded to the case or control status of participants?			NR
12. Were key potential confounding variables measured and adjusted statistically in the analyses? If matching was used, did the investigators account for matching during study analysis?		x	

Criteria Antenatal Maternal Antidepressants Drugs Treatment Affects S100B Levels in Maternal-Fetal Biological Fluids in a Dose Dependent Manner	Yes	No	Other (CD, NR, NA)*
1. Was the research question or objective in this paper clearly stated?	x		
2. Was the study population clearly specified and defined?	x		
3. Was the participation rate of eligible persons at least 50%?	x		
4. Were all the subjects selected or recruited from the same or similar populations (including the same time period)? Were inclusion and exclusion criteria for being in the study prespecified and applied uniformly to all participants?	x		
5. Was a sample size justification, power description, or variance and effect estimates provided?	x		
6. For the analyses in this paper, were the exposure(s) of interest measured prior to the outcome(s) being measured?	x		
7. Was the timeframe sufficient so that one could reasonably expect to see an association between exposure and outcome if it existed?	x		
8. For exposures that can vary in amount or level, did the study examine different levels of the exposure as related to the outcome (e.g., categories of exposure, or exposure measured as continuous variable)?	x		
9. Were the exposure measures (independent variables) clearly defined, valid, reliable, and implemented consistently across all study participants?	x		
10. Was the exposure(s) assessed more than once over time?	x		
11. Were the outcome measures (dependent variables) clearly defined, valid, reliable, and implemented consistently across all study participants?	x		
12. Were the outcome assessors blinded to the exposure status of participants?		x	
13. Was loss to follow-up after baseline 20% or less?	x		
14. Were key potential confounding variables measured and adjusted statistically for their impact on the relationship between exposure(s) and outcome(s)?	x		

4. Quality assessment – smoking

Criteria Second trimester amniotic fluid adiponectin level is affected by maternal tobacco exposure, insulin, and PAPP-A level	Yes	No	Other (CD, NR, NA)*
1. Was the research question or objective in this paper clearly stated?	x		
2. Was the study population clearly specified and defined?	x		
3. Was the participation rate of eligible persons at least 50%?	x		
4. Were all the subjects selected or recruited from the same or similar populations (including the same time period)? Were inclusion and exclusion criteria for being in the study prespecified and applied uniformly to all participants?	x		
5. Was a sample size justification, power description, or variance and effect estimates provided?		x	
6. For the analyses in this paper, were the exposure(s) of interest measured prior to the outcome(s) being measured?		x	
7. Was the timeframe sufficient so that one could reasonably expect to see an association between exposure and outcome if it existed?		x	
8. For exposures that can vary in amount or level, did the study examine different levels of the exposure as related to the outcome (e.g., categories of exposure, or exposure measured as continuous variable)?		x	
9. Were the exposure measures (independent variables) clearly defined, valid, reliable, and implemented consistently across all study participants?	x		
10. Was the exposure(s) assessed more than once over time?		x	
11. Were the outcome measures (dependent variables) clearly defined, valid, reliable, and implemented consistently across all study participants?	x		
12. Were the outcome assessors blinded to the exposure status of participants?	x		
13. Was loss to follow-up after baseline 20% or less?	x		
14. Were key potential confounding variables measured and adjusted statistically for their impact on the relationship between exposure(s) and outcome(s)?	x		

Criteria Low-level maternal exposure to nicotine associates with significant metabolic perturbations in second-trimester amniotic fluid	Yes	No	Other (CD, NR, NA)*
1. Was the research question or objective in this paper clearly stated?	x		
2. Was the study population clearly specified and defined?	x		
3. Was the participation rate of eligible persons at least 50%?			CD
4. Were all the subjects selected or recruited from the same or similar populations (including the same time period)? Were inclusion and exclusion criteria for being in the study prespecified and applied uniformly to all participants?	x		
5. Was a sample size justification, power description, or variance and effect estimates provided?		x	
6. For the analyses in this paper, were the exposure(s) of interest measured prior to the outcome(s) being measured?	x		
7. Was the timeframe sufficient so that one could reasonably expect to see an association between exposure and outcome if it existed?		x	
8. For exposures that can vary in amount or level, did the study examine different levels of the exposure as related to the outcome (e.g., categories of exposure, or exposure measured as continuous variable)?	x		
9. Were the exposure measures (independent variables) clearly defined, valid, reliable, and implemented consistently across all study participants?	x		
10. Was the exposure(s) assessed more than once over time?		x	
11. Were the outcome measures (dependent variables) clearly defined, valid, reliable, and implemented consistently across all study participants?	x		
12. Were the outcome assessors blinded to the exposure status of participants?			NR
13. Was loss to follow-up after baseline 20% or less?			NA
14. Were key potential confounding variables measured and adjusted statistically for their impact on the relationship between exposure(s) and outcome(s)?	x		

Criteria Influence of Maternal Smoking during Pregnancy on Oxidant Status in Amniotic Fluid	Yes	No	Other (CD, NR, NA)*
1. Was the research question or objective in this paper clearly stated?	x		
2. Was the study population clearly specified and defined?	x		
3. Was the participation rate of eligible persons at least 50%?			CD
4. Were all the subjects selected or recruited from the same or similar populations (including the same time period)? Were inclusion and exclusion criteria for being in the study prespecified and applied uniformly to all participants?	x		
5. Was a sample size justification, power description, or variance and effect estimates provided?		x	
6. For the analyses in this paper, were the exposure(s) of interest measured prior to the outcome(s) being measured?	x		
7. Was the timeframe sufficient so that one could reasonably expect to see an association between exposure and outcome if it existed?		x	
8. For exposures that can vary in amount or level, did the study examine different levels of the exposure as related to the outcome (e.g., categories of exposure, or exposure measured as continuous variable)?		x	
9. Were the exposure measures (independent variables) clearly defined, valid, reliable, and implemented consistently across all study participants?	x		
10. Was the exposure(s) assessed more than once over time?		x	
11. Were the outcome measures (dependent variables) clearly defined, valid, reliable, and implemented consistently across all study participants?	x		
12. Were the outcome assessors blinded to the exposure status of participants?			NR
13. Was loss to follow-up after baseline 20% or less?			NA
14. Were key potential confounding variables measured and adjusted statistically for their impact on the relationship between exposure(s) and outcome(s)?		x	

5. Quality assessment – other

Criteria ***Circulating and Placental Growth-Differentiation Factor 15 in Preeclampsia and in Pregnancy Complicated by Diabetes Mellitus***	Yes	No	Other (CD, NR, NA)*
1. Was the research question or objective in this paper clearly stated?	x		
2. Was the study population clearly specified and defined?	x		
3. Was the participation rate of eligible persons at least 50%?			CD
4. Were all the subjects selected or recruited from the same or similar populations (including the same time period)? Were inclusion and exclusion criteria for being in the study prespecified and applied uniformly to all participants?	x		
5. Was a sample size justification, power description, or variance and effect estimates provided?		x	
6. For the analyses in this paper, were the exposure(s) of interest measured prior to the outcome(s) being measured?		x	
7. Was the timeframe sufficient so that one could reasonably expect to see an association between exposure and outcome if it existed?		x	
8. For exposures that can vary in amount or level, did the study examine different levels of the exposure as related to the outcome (e.g., categories of exposure, or exposure measured as continuous variable)?		x	
9. Were the exposure measures (independent variables) clearly defined, valid, reliable, and implemented consistently across all study participants?	x		
10. Was the exposure(s) assessed more than once over time?		x	
11. Were the outcome measures (dependent variables) clearly defined, valid, reliable, and implemented consistently across all study participants?	x		
12. Were the outcome assessors blinded to the exposure status of participants?			NR
13. Was loss to follow-up after baseline 20% or less?			NA
14. Were key potential confounding variables measured and adjusted statistically for their impact on the relationship between exposure(s) and outcome(s)?		x	

Criteria Neuron specific enolase in amniotic fluid — a possible indicator for fetal distress and brain implication	Yes	No	Other (CD, NR, NA)*
1. Was the research question or objective in this paper clearly stated?	x		
2. Was the study population clearly specified and defined?	x		
3. Was the participation rate of eligible persons at least 50%?			CD
4. Were all the subjects selected or recruited from the same or similar populations (including the same time period)? Were inclusion and exclusion criteria for being in the study prespecified and applied uniformly to all participants?	x		
5. Was a sample size justification, power description, or variance and effect estimates provided?		x	
6. For the analyses in this paper, were the exposure(s) of interest measured prior to the outcome(s) being measured?		x	
7. Was the timeframe sufficient so that one could reasonably expect to see an association between exposure and outcome if it existed?		x	
8. For exposures that can vary in amount or level, did the study examine different levels of the exposure as related to the outcome (e.g., categories of exposure, or exposure measured as continuous variable)?	x		
9. Were the exposure measures (independent variables) clearly defined, valid, reliable, and implemented consistently across all study participants?	x		
10. Was the exposure(s) assessed more than once over time?		x	
11. Were the outcome measures (dependent variables) clearly defined, valid, reliable, and implemented consistently across all study participants?	x		
12. Were the outcome assessors blinded to the exposure status of participants?			NR
13. Was loss to follow-up after baseline 20% or less?			NA
14. Were key potential confounding variables measured and adjusted statistically for their impact on the relationship between exposure(s) and outcome(s)?		x	

Criteria Decrease in 3-nitrotyrosine in the amniotic fluid of women with cytomegalovirus infection	Yes	No	Other (CD, NR, NA)*
1. Was the research question or objective in this paper clearly stated?	x		
2. Was the study population clearly specified and defined?	x		
3. Was the participation rate of eligible persons at least 50%?			CD
4. Were all the subjects selected or recruited from the same or similar populations (including the same time period)? Were inclusion and exclusion criteria for being in the study prespecified and applied uniformly to all participants?	x		
5. Was a sample size justification, power description, or variance and effect estimates provided?		x	
6. For the analyses in this paper, were the exposure(s) of interest measured prior to the outcome(s) being measured?		x	
7. Was the timeframe sufficient so that one could reasonably expect to see an association between exposure and outcome if it existed?		x	
8. For exposures that can vary in amount or level, did the study examine different levels of the exposure as related to the outcome (e.g., categories of exposure, or exposure measured as continuous variable)?	x		
9. Were the exposure measures (independent variables) clearly defined, valid, reliable, and implemented consistently across all study participants?	x		
10. Was the exposure(s) assessed more than once over time?		x	
11. Were the outcome measures (dependent variables) clearly defined, valid, reliable, and implemented consistently across all study participants?	x		
12. Were the outcome assessors blinded to the exposure status of participants?			NR
13. Was loss to follow-up after baseline 20% or less?			NA
14. Were key potential confounding variables measured and adjusted statistically for their impact on the relationship between exposure(s) and outcome(s)?	x		

Criteria Maternal Obesity Affects Fetal Neurodevelopmental and Metabolic Gene Expression: A Pilot Study	Yes	No	Other (CD, NR, NA)*
1. Was the research question or objective in this paper clearly stated?	x		
2. Was the study population clearly specified and defined?	x		
3. Was the participation rate of eligible persons at least 50%?			NR
4. Were all the subjects selected or recruited from the same or similar populations (including the same time period)? Were inclusion and exclusion criteria for being in the study prespecified and applied uniformly to all participants?	x		
5. Was a sample size justification, power description, or variance and effect estimates provided?	x		
6. For the analyses in this paper, were the exposure(s) of interest measured prior to the outcome(s) being measured?	x		
7. Was the timeframe sufficient so that one could reasonably expect to see an association between exposure and outcome if it existed?	x		
8. For exposures that can vary in amount or level, did the study examine different levels of the exposure as related to the outcome (e.g., categories of exposure, or exposure measured as continuous variable)?	x		
9. Were the exposure measures (independent variables) clearly defined, valid, reliable, and implemented consistently across all study participants?	x		
10. Was the exposure(s) assessed more than once over time?		x	
11. Were the outcome measures (dependent variables) clearly defined, valid, reliable, and implemented consistently across all study participants?	x		
12. Were the outcome assessors blinded to the exposure status of participants?			NR
13. Was loss to follow-up after baseline 20% or less?			NA
14. Were key potential confounding variables measured and adjusted statistically for their impact on the relationship between exposure(s) and outcome(s)?		x	

Criteria Second trimester amniotic fluid glucose, uric acid, phosphate, potassium, and sodium concentrations in relation to maternal pre-pregnancy BMI and birth weight centiles	Yes	No	Other (CD, NR, NA)*
1. Was the research question or objective in this paper clearly stated?	x		
2. Was the study population clearly specified and defined?	x		
3. Was the participation rate of eligible persons at least 50%?			NR
4. Were all the subjects selected or recruited from the same or similar populations (including the same time period)? Were inclusion and exclusion criteria for being in the study prespecified and applied uniformly to all participants?	x		
5. Was a sample size justification, power description, or variance and effect estimates provided?	x		
6. For the analyses in this paper, were the exposure(s) of interest measured prior to the outcome(s) being measured?	x		
7. Was the timeframe sufficient so that one could reasonably expect to see an association between exposure and outcome if it existed?	x		
8. For exposures that can vary in amount or level, did the study examine different levels of the exposure as related to the outcome (e.g., categories of exposure, or exposure measured as continuous variable)?	x		
9. Were the exposure measures (independent variables) clearly defined, valid, reliable, and implemented consistently across all study participants?	x		
10. Was the exposure(s) assessed more than once over time?		x	
11. Were the outcome measures (dependent variables) clearly defined, valid, reliable, and implemented consistently across all study participants?	x		
12. Were the outcome assessors blinded to the exposure status of participants?			NR
13. Was loss to follow-up after baseline 20% or less?			NA
14. Were key potential confounding variables measured and adjusted statistically for their impact on the relationship between exposure(s) and outcome(s)?	x		
